# Four myriapod relatives – but who are sisters? No end to debates on relationships among the four major myriapod subgroups

**DOI:** 10.1186/s12862-020-01699-0

**Published:** 2020-11-04

**Authors:** Nikolaus U. Szucsich, Daniela Bartel, Alexander Blanke, Alexander Böhm, Alexander Donath, Makiko Fukui, Simon Grove, Shanlin Liu, Oliver Macek, Ryuichiro Machida, Bernhard Misof, Yasutaka Nakagaki, Lars Podsiadlowski, Kaoru Sekiya, Shigekazu Tomizuka, Björn M. Von Reumont, Robert M. Waterhouse, Manfred Walzl, Guanliang Meng, Xin Zhou, Günther Pass, Karen Meusemann

**Affiliations:** 1 Department of Evolutionary Biology, University of Vienna, A-1090 Vienna, Austria; 2 Central Research Laboratories, Natural History Museum of Vienna, A-1010 Vienna, Austria; 3 Institute for Zoology, Biocenter, University of Cologne, D-50674 Cologne, Germany; 4 Institute of Evolutionary Biology and Animal Ecology, University of Bonn, D-53121 Bonn, Germany; 5 Centre for Molecular Biodiversity Research, Zoological Research Museum Alexander Koenig, D-53113 Bonn, Germany; 6 Department of Biology, Graduate School of Science and Engineering, Ehime University, Bunkyo-cho, Matsuyama, Ehime 790-8577 Japan; 7 Invertebrate Zoology, Collections and Research Facility, Tasmanian Museum and Art Gallery, Rosny, Tasmania 7018 Australia; 8 Department of Entomology, China Agricultural University, Beijing, 100193 People’s Republic of China; 9 Sugadaira Research Station, Mountain Science Center, University of Tsukuba, Sugadaira, Ueda, Nagano, 386-2204 Japan; 10 Zoological Research Museum Alexander Koenig, D-53113 Bonn, Germany; 11 Matsunoyamamatsuguchi, Tokamachi, Niigata, 942-1411 Japan; 12 LOEWE Centre for Translational Biodiversity Genomics (LOEWE TBG), Senckenberganlage 25, 60325 Frankfurt, Germany; 13 Animal Venomics, Institute for Insect Biotechnology, University of Giessen, Heinrich Buff Ring 26-32, D-35394 Giessen, Germany; 14 Department of Ecology and Evolution, University of Lausanne and Swiss Institute of Bioinformatics, 1015 Lausanne, Switzerland; 15 Centre of Taxonomy and Evolutionary Research, Zoological Research Museum Alexander Koenig, D-53113 Bonn, Germany; 16 Department of Evolutionary Biology and Ecology, Institute of Biology I (Zoology), University of Freiburg, D-79104 Freiburg, Germany; 17 Australian National Insect Collection, National Research Collections Australia, CSIRO, ACT, Canberra, 2601 Australia

**Keywords:** Internal rooting, Phylogenetics, Arthropod phylogeny, Quartet topology, Conflict, Confounding signal, Transcriptomes, RNA-Seq, Phylogenomics

## Abstract

**Background:**

Phylogenetic relationships among the myriapod subgroups Chilopoda, Diplopoda, Symphyla and Pauropoda are still not robustly resolved. The first phylogenomic study covering all subgroups resolved phylogenetic relationships congruently to morphological evidence but is in conflict with most previously published phylogenetic trees based on diverse molecular data. Outgroup choice and long-branch attraction effects were stated as possible explanations for these incongruencies. In this study, we addressed these issues by extending the myriapod and outgroup taxon sampling using transcriptome data.

**Results:**

We generated new transcriptome data of 42 panarthropod species, including all four myriapod subgroups and additional outgroup taxa. Our taxon sampling was complemented by published transcriptome and genome data resulting in a supermatrix covering 59 species. We compiled two data sets, the first with a full coverage of genes per species (292 single-copy protein-coding genes), the second with a less stringent coverage (988 genes). We inferred phylogenetic relationships among myriapods using different data types, tree inference, and quartet computation approaches. Our results unambiguously support monophyletic Mandibulata and Myriapoda. Our analyses clearly showed that there is strong signal for a single unrooted topology, but a sensitivity of the position of the internal root on the choice of outgroups. However, we observe strong evidence for a clade Pauropoda+Symphyla, as well as for a clade Chilopoda+Diplopoda.

**Conclusions:**

Our best quartet topology is incongruent with current morphological phylogenies which were supported in another phylogenomic study. AU tests and quartet mapping reject the quartet topology congruent to trees inferred with morphological characters. Moreover, quartet mapping shows that confounding signal present in the data set is sufficient to explain the weak signal for the quartet topology derived from morphological characters. Although outgroup choice affects results, our study could narrow possible trees to derivatives of a single quartet topology. For highly disputed relationships, we propose to apply a series of tests (AU and quartet mapping), since results of such tests allow to narrow down possible relationships and to rule out confounding signal.

## Background

With about 15,000 described extant species, myriapods are a diverse group of terrestrial arthropods [1]. Myriapod monophyly is currently uncontested and four major subgroups are recognised: the species-rich Chilopoda (centipedes) and Diplopoda (millipedes), and the much less speciose Pauropoda and Symphyla. Phylogenomic data from myriapods are still scarce, especially pauropods and symphylans are highly understudied. The first phylogenomic study that included all four subgroups supported monophyletic Myriapoda and the monophyly of each major subgroup [2]. However, regarding the relationships among these four subgroups, the inferred tree was incongruent with all previous molecular phylogenies, instead agreeing with trees inferred from morphological data supporting a sister group relationship of Diplopoda+Pauropoda. This millipede-pauropod group is known as Dignatha, sharing modified mouthparts, due to the lack of appendage buds on the second maxillary segment. Symphyla were proposed as sister to Dignatha, supporting monophyletic Progoneata (Diplopoda+Pauropoda+Symphyla) based on the position of their genital apertures near the anterior end of the trunk (for a review see [3]). Fernandez and colleagues [2] greatly increased the amount of available data for phylogenomic analyses. At the same time, the authors likewise emphasised a strong dependence of results on the choice of outgroups.

To address relationships of the four myriapod subgroups, we generated new myriapod RNA-Seq data from 42 species that we combined with published data: Using data from a total of 59 species, we compiled and analysed two phylogenomic data sets covering the four myriapod subgroups, hexapods, crustaceans, chelicerates and onychophorans (velvet worms) (Table 1), one including 292 genes (maximal gene coverage) and the other including 988 genes (relaxed setting). Our resulting trees and alternative hypotheses were subjected to two tests: approximate unbiased (AU) tests [13] and Four-cluster Likelihood-Mapping (FcLM) [14]. Additionally, we explored potential confounding signal that might bias tree inference by a FcLM permutation approach (for the rationale see e.g., [10, 15, 16]). All tests were performed to narrow down the number of possible topologies (and trees).

**Table 1:** Species included in this study. Species marked with * are included in the ortholog set. *Zootermopsis* ($) was excluded from the analyses after orthology assignment. BioProject accession numbers refer to NCBI BioProject database, included in the Umbrella project “The 1KITE project: Evolution of insects”. OGS: official gene sets from available genomes. For references, please refer to the main text and Additional File 1. Details e.g., accession numbers, collecting information, data sources are provided in Additional File 2-Table S1-S5

Taxonomy	***Genus, species***	BioProject accession numbers / OGS	Source, study / project
Onychophora, Peripatopsidae	*Peripatopsis capensis*	PRJNA236598	[4]
Onychophora, Peripatopsidea	*Peripatoides novaezealandiae*	PRJNA316414	this study (VIEART)
Chelicerata, Amblypygi	*Damon diadema*	PRJNA316401	this study (VIEART)
Chelicerata, Arachnida, Acari	Ixodes scapularis*	OGS 1.3 (Wikl OGS)	[5, 6]
Chelicerata, Arachnida, Acari	*Archegozetes longisetosus*	PRJNA254245	this study (1KITE)
Chelicerata, Arachnida, Araneae	*Araneus diadematus*	PRJNA316396	this study (VIEART)
Chelicerata, Arachnida, Opiliones	*Egaenus convexus*	PRJNA316402	this study (VIEART)
Chelicerata, Arachnida, Scorpiones	*Euscorpius sicanus*	PRJNA254264	this study (1KITE)
Chelicerata, Pygnogonida	*Nymphon gracile*	PRJNA254293	this study (1KITE)
Myriapoda, Chilopoda, Craterostigmomorpha	*Craterostigmus tasmanianus*	PRJNA299165	this study (1KITE)
Myriapoda, Chilopoda, Geophilomorpha	*Henia illyrica*	PRJNA316408	this study (VIEART)
Myriapoda, Chilopoda, Geophilomorpha	*Clinopodes flavidus*	PRJNA254253	this study (1KITE)
Myriapoda, Chilopoda, Geophilomorpha	*Himantarium gabrielis*	PRJNA254270	this study (1KITE)
Myriapoda, Chilopoda, Geophilomorpha	Strigamia maritima*	OGS 1.22	[7]
Myriapoda, Chilopoda, Geophilomorpha	*Strigamia acuminata*	PRJNA316419	this study (VIEART)
Myriapoda, Chilopoda, Geophilomorpha	*Schendyla carniolensis*	PRJNA316418	this study (VIEART)
Myriapoda, Chilopoda, Lithobiomorpha	*Eupolybothrus cavernicolus*	PRJEB4548	[8]
Myriapoda, Chilopoda, Lithobiomorpha	*Eupolybothrus fasciatus*	PRJNA316403	this study (VIEART)
Myriapoda, Chilopoda, Lithobiomorpha	*Eupolybothrus tridentinus*	PRJNA316404	this study (VIEART)
Myriapoda, Chilopoda, Lithobiomorpha	*Lithobius forficatus*	PRJNA254283	this study (1KITE)
Myriapoda, Chilopoda, Scolopendromorpha	*Cryptops anomalans*	PRJNA316400	this study (VIEART)
Myriapoda, Chilopoda, Scolopendromorpha	*Cryptops hortensis*	PRJNA237130	[4]
Myriapoda, Chilopoda, Scolopendromorpha	*Scolopendra cingulata*	PRJNA254307	this study (1KITE)
Myriapoda, Chilopoda, Scolopendromorpha	*Scolopocryptops rubiginosus*	PRJNA254308	this study (1KITE)
Myriapoda, Chilopoda, Scutigeromorpha	*Scutigera coleoptrata*	PRJNA254309	this study (1KITE)
Myriapoda, Diplopoda, Callipodida	*Callipus foetidissimus*	PRJNA316397	this study (VIEART)
Myriapoda, Diplopoda, Chordeumatida	*Craspedosoma* sp.	PRJNA316399	this study (VIEART)
Myriapoda, Diplopoda, Glomerida	*Haploglomeris multistriata*	PRJNA316407	this study (VIEART)
Myriapoda, Diplopoda, Glomerida	*Glomeridella minima*	PRJNA316405	this study (VIEART)
Myriapoda, Diplopoda, Julida	*Ommatoiulus sabulosus*	PRJNA254294	this study (1KITE)
Myriapoda, Diplopoda, Julida	*Thalassisobates littoralis*	PRJNA254314	this study (1KITE)
Myriapoda, Diplopoda, Polydesmida	*Polydesmus complanatus*	PRJNA316415	this study (VIEART)
Myriapoda, Diplopoda, Polyxenida	*Eudigraphis takakuwai*	PRJNA254263	this study (1KITE)
Myriapoda, Diplopoda, Polyxenida	*Polyxenus lagurus*	PRJNA316416	this study (VIEART)
Myriapoda, Diplopoda, Polyzoniida	*Polyzonium germanicum*	PRJNA316417	this study (VIEART)
Myriapoda, Pauropoda	*Acopauropus ornatus*	PRJNA316395	this study (VIEART)
Myriapoda, Symphyla	*Symphylella* sp.	PRJNA254313	this study (1KITE)
Myriapoda, Symphyla	*Hanseniella nivea*	PRJNA316406	this study (VIEART)
Myriapoda, Symphyla	*Hanseniella* sp.	PRJNA254267	this study (1KITE)
Crustacea, Branchiopoda, Diplostraca	Daphnia pulex*	OGS 1.22	[9]
Crustacea, Branchiopoda, Anostraca	*Eubranchipus grubii*	PRJNA254262	this study (1KITE)
Crustacea, Branchiopoda, Notostraca	*Triops cancriformis*	PRJNA254320	this study (1KITE)
Crustacea, Malacostraca, Leptostraca	*Nebalia bipes*	PRJNA254287	this study (1KITE)
Crustacea, Malacostraca, Syncarida	*Anaspides tasmaniae*	PRJNA254244	this study (1KITE)
Crustacea, Maxillopoda, Copepoda	*Hemidiaptomus amblyodon*	PRJNA254268	this study (1KITE)
Crustacea, Maxillopoda, Copepoda	*Tisbe furcata*	PRJNA254316	this study (1KITE)
Crustacea, Ostracoda, Myodocopida	*Vargula hilgendorfii*	PRJNA274392	this study (1KITE)
Crustacea, Remipedia, Nectiopoda	*Xibalbanus tulumensis*	PRJNA254312	this study (1KITE)
Hexapoda, Protura	*Acerentomon maius*	PRJNA219521	[10]; Current assembly: [11]
Hexapoda, Diplura	*Occasjapyx japonicus*	PRJNA286654	[10]; Current assembly: [11]
Hexapoda, Collembola, Entomobryomorpha	*Pogonognathellus* sp.	PRJNA219595	[10]; Current assembly: [11]
Hexapoda, Collembola, Poduromorpha	*Anurida maritima*	PRJNA219523	[10]; Current assembly: [11]
Hexapoda, Archaeognatha	*Machilis hrabei*	PRJNA219574	[10]; Current assembly: [11]
Hexapoda, Zygentoma	*Atelura formicaria*	PRJNA219527	[10]; Current assembly: [11]
Hexapoda, Odonata	*Ladona fulva*	OGS 0.5.3	i5K, unpublished, Kindly provided by the i5K Consortium
Hexapoda, Ephemeroptera	Ephemera danica*	OGS 0.5.3	i5K, unpublished, Kindly provided by the i5K Consortium
Hexapoda, Blattodea	Zootermopsis nevadensis*,$	OGS 2.2	[12]
Hexapoda, Blattodea	*Periplaneta americana*	PRJNA219590	[10]; Current assembly: [11]
Hexapoda, Hemiptera	*Essigella californica*	PRJNA219554	[10]; Current assembly: [11]
Hexapoda, Raphidioptera	*Xanthostigma xanthostigma*	PRJNA219617	[10]; Current assembly: [11]

**Fig. 1:**
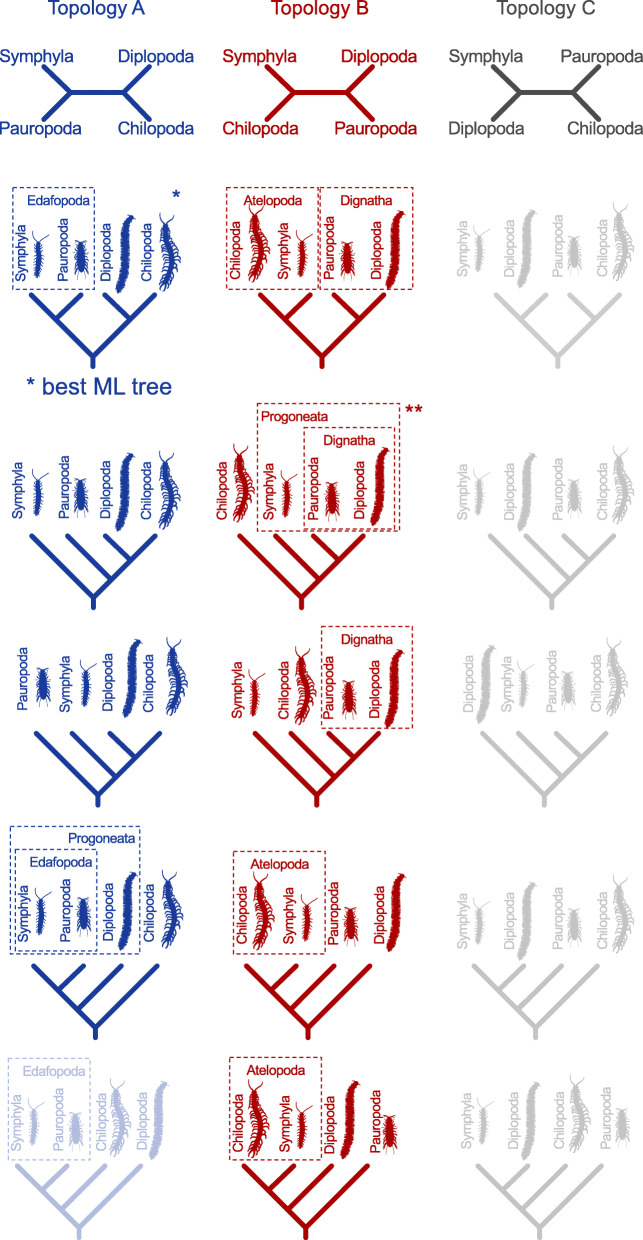
Hypotheses on relationships of the major myriapod lineages Chilopoda, Diplopoda, Symphyla and Pauropoda. Quartet topology A (in blue): Pauropoda+Symphyla and Chilopoda+Diplopoda. The column displays all trees that can be derived from this quartet topology by different internal rooting. *: best ML tree of our study. Quartet topology B (in red): Chilopoda+Symphyla and Diplopoda+Pauropoda. The column displays all trees that can be derived from this quartet topology by different internal rooting. **: Main ML tree inferred by Fernandez and co-authors [2] and preferred morphological tree. Quartet topology **c** (in grey): Diplopoda+Symphyla and Chilopoda+Pauropoda. The column displays all trees that can be derived from this quartet topology by different internal rooting, yet none of them is supported by any study

For each quartet of taxa, three fully resolved unrooted topologies exist. From each of these three topologies, five possible trees can be derived that differ only in the placement of the internal root (Fig. 1, columns A, B and C). Alternative trees either (i) may be derived by differential rooting of the same quartet topology (Fig. 1, trees within a column), or (ii) may be derivatives of different topologies (Fig. 1, trees among different columns). The first case only differs in character polarisation while the second case indicates incongruences between topologies.

The tree proposed by Fernandez and colleagues [2] (Fig. 1, marked with **) is congruent with an unrooted quartet topology with Diplopoda+Pauropoda and Chilopoda+Symphyla (Fig. 1, quartet topology B). Of all published phylogenies inferred from molecular sequence data, only trees of [17, 18] are also congruent with quartet topology B. All other published phylogenies [19–22], can be derived from the quartet topology with Pauropoda+Symphyla and Chilopoda+Diplopoda (Fig. 1, quartet topology A). Fernandez and colleagues [2] argued, that the support for Edafopoda (Pauropoda+Symphyla) in previous studies could be explained by artefacts, especially long-branch attraction of Pauropoda towards the equally long-branched Pancrustacea (crustaceans and hexapods), introduced when the latter were included as an outgroup. We further tested the dependence of the inferred relationships on outgroup choice, and whether preferred phylogenetic signal from transcriptome data differs from other published molecular data sets, as suggested by Fernandez and colleagues [2].

## Results

### From sequencing to informative data sets and tree inference

After sequencing, de novo assembly, and cleaning of transcripts (see Additional File 1), on average more than 80% of our ortholog set (comprising 2716 single-copy protein-coding genes or ortholog groups, OGs) were identified per sample (details in Additional File 1, Additional File 2-Table S6). Alignment, alignment refinement, removal of outlier sequences, identification and removal of ambiguously aligned sections, concatenation of gene partitions and optimisation of the data set by removal of gene partitions lacking putative information content, resulted in two data sets:

(i) the STRICT data set for which each gene partition was represented by each of the 59 species, thus resulting in a 100% coverage of all gene partitions, included 292 gene partitions on amino-acid level and spanned a length of 95,797 aligned sites on amino acid level (overall information content (IC): 0.30, alignment completeness score 82.53%).
(ii) the RELAXED data set for which each gene partition was represented by at least one species of each selected group (Additional File 2-Table S7), included 988 gene partitions on amino-acid level spanning a superalignment length of 348,917 sites (overall IC 0.27, alignment completeness score 72.13%). Supermatrix diagnostics are provided in Additional File 1, Additional File 2-Table S8 and Additional File 3.

Both data sets displayed heterogeneity across lineages and rejecting stationary, (time-)reversible and homogeneous (SRH) conditions ([23, 24], Additional File 1 and Additional File 3-Fig. S1).

In the corresponding nucleotide data matrices, only the second codon positions were retained as data violating the least the SRH conditions.

After selecting the best partition schemes and best-fitting substitution models per partition, we found all inferred Maximum-Likelihood (ML) trees to be similar, first comparing all ML trees inferred for each data set separately and then comparing all ML trees across all data sets. This outcome was found irrespective of analysed data type - amino acid (aa) or nucleotide (nt) level - and whether the partitioned or unpartitioned approach with the CAT-like protein mixture model was applied [25, 26] (details are provided in Additional File 1). The only minor exception concerned the sister group of Geophilomorpha (RELAXEDaa data set) resulting in two possible trees (Additional File 1). Convergence of bootstrap replicates [27] was always fulfilled, and all our data sets were free of rogue taxa [28].

### Phylogenetic relationships and identification of conflicts

All analyses performed on the STRICT and RELAXED data sets including the full taxon sampling showed the same outcome with respect to the three main questions of the present study: (i) Myriapoda are monophyletic, (ii) Myriapoda are the sister group to Pancrustacea, and (iii) there is a high support for the quartet topology with Pauropoda+Symphyla and Chilopoda+Diplopoda. These results were consistently recovered, irrespective of data type (i.e. aa or nt) (Additional File 2-Table S7).

#### (i & ii) Myriapoda and placement within arthropods

All our analyses retrieved Myriapoda as the monophyletic sister group of Pancrustacea, unambiguously supporting Mandibulata (the name refers to the jawlike first pair of mouthparts, the mandibles, present in myriapods, crustaceans and hexapods). Our FcLM analyses with Pancrustacea, Myriapoda, Chelicerata and velvet worms (Onychophora) as the four-taxon set showed a strong preference for Myriapoda+Pancrustacea, a result fully congruent with all inferred ML trees (Additional File 2-Table S9 and Additional File 3-Figs. S7-S17). The support for Mandibulata cannot be explained by confounding signal, neither by compositional and among-lineage heterogeneity nor by non-randomly distributed data (details in Additional Files 1 and 2).

(iii) *Relationships among the four myriapod subgroups*

**Fig. 2:**
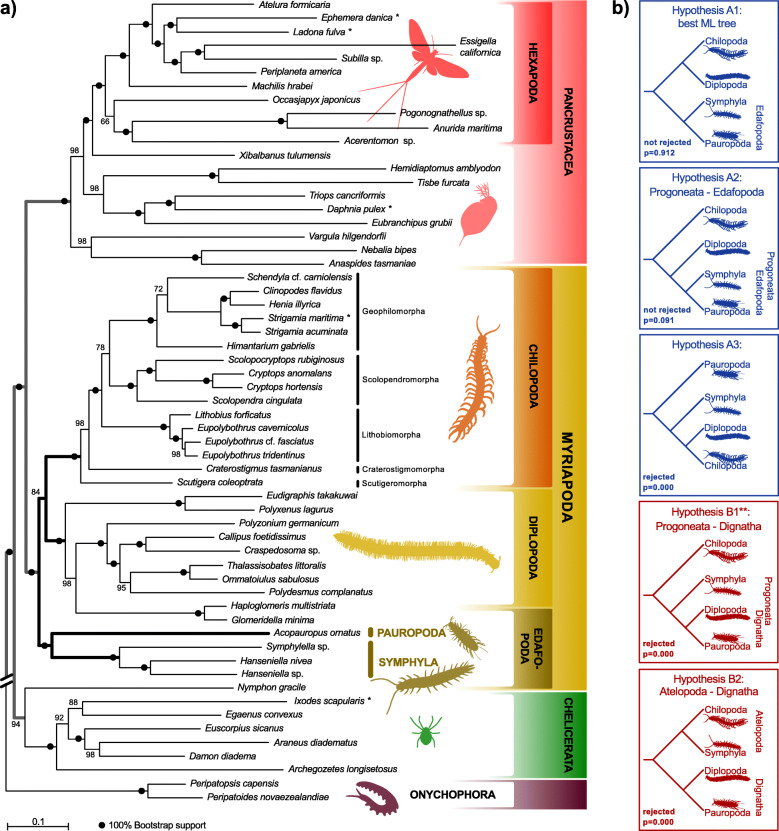
Inferred myriapod phylogenetic relationships tested with the Approximate unbiased (AU) test. **a** best Maximum-Likelihood tree inferred with IQ-TREE derived from our STRICTaa dataset (59 taxa, alignment length: 95,797 amino acid positions, 292 gene partitions). This tree was also supported by various other datasets in our study. Statistical support was derived from 100 non-parametric bootstrap replicates. The tree was rooted with Onychophora. Maximal statistical support is indicated with a black dot, support is furthermore displayed in numbers (%) when not maximal. **b** Results of the approximate unbiased (AU) test on the STRICT data set on amino acid level. Displayed in blue are trees that can be derived from quartet topology A, displayed in red are trees that can be derived from quartet topology B (Fig. 1). Hypothesis A1 (identical with our best ML tree) and A2 were not rejected, all other trees were significantly rejected (*p* < 0.05). ^$^: Note that we had two variants of Hypothesis B1 that differed by the placement of Scolopendromorpha, Lithobiomorpha and Geophilomorpha within centipedes

**Fig. 3:**
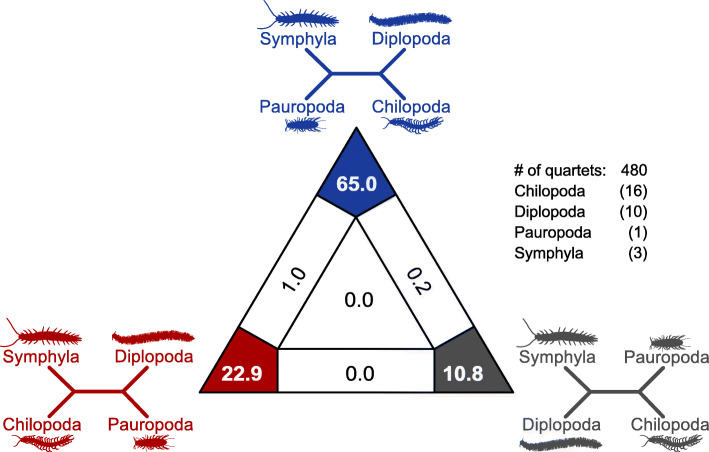
Four-cluster Likelihood-Mapping results on myriapod phylogenetic relationships. Quartet proportions (in %) mapped on a 2D-simplex graph supporting different quartet topologies. In parentheses are given the number of included species of the respective myriapod subgroup (Additional File 2-Table S10). The majority of all drawn quartets (480 quartets) support quartet topology A (Figs. 1 and 2) while quartet topology B and C received support by only a small proportion of all quartets. In contrast to quartet topology A, quartet support for quartet topology B and C was small and could be fully explained by confounding signal (Table 2)

Our analyses always revealed a sister group relationship of Pauropoda+Symphyla (coined Edafopoda by [20]) with strong bootstrap and transfer bootstrap support, and a sister group relationship of Chilopoda+Diplopoda with moderate statistical support. A sister group relationship of Pauropoda+Symphyla, and Chilopoda+Diplopoda, respectively, was not rejected by AU tests (Fig. 1, quartet topology A and Fig. 2a, b). However, Diplopoda as sister group to Edafopoda supporting Progoneata was also not rejected. Quartet topology B (Fig. 1) with Dignatha (i.e. Diplopoda+Pauropoda) as, for instance, inferred by Fernandez and colleagues [2], was rejected, irrespective of whether the sister group of Dignatha was Chilopoda, Symphyla, or a clade Chilopoda+Symphyla. This was also independent of the internal relationships among chilopod subgroups. FcLM of the four myriapod subgroups resulted in strong support for the unrooted quartet topology with Chilopoda+Diplopoda and Pauropoda+Symphyla (quartet topology A; Fig. 3; Table 2). This quartet topology is congruent with five possible trees, including our best ML tree (Fig. 1, quartet topology A marked with * and Fig. 2a, b). Again, this result could not be explained by confounding signal, as shown by the FcLM on permuted data sets (Additional File 1 and Additional File 2-Table S11). In contrast, about one fifth of all drawn quartets supported Diplopoda+Pauropoda and Chilopoda+Symphyla (quartet topology B, Fig. 1). However, the support for this quartet topology – congruent with the tree proposed by Fernandez and colleagues [2] – can be fully explained by confounding signal, i.e. by heterogeneity among lineages violating SRH conditions and by non-randomly distributed data (Additional File 1 and Additional File 2-Table S11, permutation approaches) in our STRICT amino acid data set.

**Table 2:** Four-cluster Likelihood-Mapping results among the four major myriapod subgroups. Data set STRICTaa (95,797 alignment sites, 292 gene partitions, merged into 215 meta-partitions). # of drawn quartets: 480. Cluster 1: Chilopoda (Chil), Cluster 2: Diplopoda (Dipl), Cluster 3: Pauropoda (Paur), Cluster 4: Symphyla (Sym). Given are percentages [%] of drawn quartets that map into areas in the 2D-simplex graph (Fig. 3). Quartet topology A (in blue): unambiguous support for Chilopoda+Diplopoda and Pauropoda+Symphyla. Quartet topology B (in red): unambiguous support for Chilopoda+Symphyla and Diplopoda+Pauropoda. Quartet topology C (in grey): unambiguous support for Chilopoda+Pauropoda and Diplopoda+Symphyla. Quartets that map in other outer regions of the simplex graph are partly informative, quartets that map into the centre area are not informative. Question addressed: Is there alterative signal despite the clustering of Pauropoda+Symphyla (i.e. Edafopoda) and Chilopoda+Diplopoda (quartet topology A); can quartet topology A, B or C be explained by confounding signal?

data set	Topology A ^**a**^	Topology B ^**a**^	Topology C ^**a**^	Topology A-C	Topology B-C	Topology A-B	center area
	(Chil,Dipl) – (Paur,Sym)	(Chil,Sym) – (Dipl,Paur)	(Chil,Paur) – (Dipl,Sym)				
**original**	**65%**^**$**^	22.9%	10.8%	0.2%	0.0%	1.0%	0.0%
**permutation I**	15.6%	37.9%	34.4%	2.9%	5.6%	2.5%	1.0%
**permutation II**	20.4%	27.9%	37.9%	4.4%	5.0%	2.9%	1.5%
**permutation III**	24.6%	30.8%	30.2%	4.8%	4.6%	3.1%	1.9%

* consistent to topologies A, B and C in Fig. 1. In the IQ-TREE output corresponds Topology A = Voronoi cell 1, Topology B = Voronoi cell 3, Topology C = Voronoi cell 2, Topology A-C = Voronoi cell 4, Topology B-C = Voronoi cell 5, Topology A-B = Voronoi cell 6 and the center area refers to Voronoi cell 7. ^**$**^ largest proportion of drawn quartets in bold, see Fig. 3

#### Outgroup dependence of myriapod internal relationships

We generated two variations from our data set STRICTaa (on amino acid level) to explore a possible dependence of inferred relationships among the four myriapod subgroups on the chosen outgroup (Additional File 1, and Additional File 2-Table S10).

**Fig. 4:**
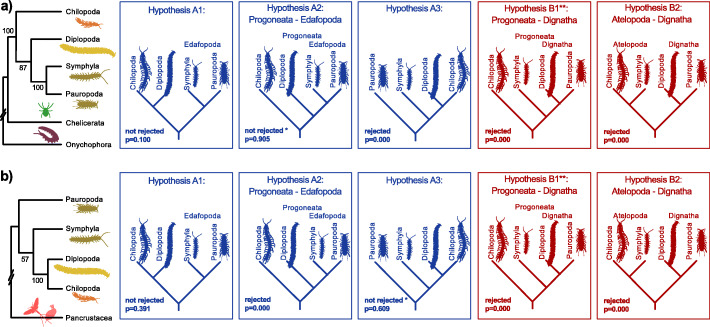
Phylogenetic relationships and outgroup dependence among the four major myriapod subgroups. **a** on the left: schematised relationships derived from ML tree inference with IQ-TREE among the myriapod subgroups when including only Chelicerata and Onychophora in STRICT amino acid data set while excluding Pancrustacea (STRICTaa_ChO). Statistical bootstrap support was inferred from 100 non-parametric bootstrap replicates; on the right: results of the AU test of five alternative trees (in blue: trees derived from quartet topology A, in red: trees derived from quartet topology B, the tree marked with ** is the tree proposed by Fernandez and colleagues [2] and supported by morphological evidence (see [3]). Note that two variants of Hypothesis B1 exist that differed by the placement of Scolopendromorpha, Lithobiomorpha and Geophilomorpha within centipedes. Hypothesis A1 and A2 (derived from quartet topology A) were not rejected while all others were rejected (p < 0.05). **b** on the left: schematised relationships derived from ML tree inference of our STRICT amino acid data set with IQ-TREE among the myriapod subgroups with Pancrustacea as the sole outgroup (Chelicerata and Onychophora excluded). Statistical bootstrap support was inferred from 100 non-parametric bootstrap replicates; on the right: results of the AU test of five alternative trees (in blue: trees derived from quartet topology A, in red: trees derived from quartet topology B (Fig. 1). **: see **a**. Hypothesis A1 and A3 (derived from quartet topology A) were not rejected while all others were rejected (p < 0.05)

The first data set, STRICTaa_ChO, included all myriapods, all chelicerates and onychophorans, excluding pancrustaceans. ML tree inference again resulted in a sister group relationship of Pauropoda and Symphyla (i.e. Edafopoda) (Fig. 4a), a derivative of the quartet topology A (Fig. 1). In contrast to the STRICT data set that comprises the full taxon sampling (Fig. 2), Diplopoda was sister to Edafopoda, thus supporting Progoneata (Fig. 4a, Hypothesis A2). To apply FcLM analyses in a test for outgroup dependence, we created four subsets; in each of them one of the four myriapod subgroups was excluded, so that three myriapod subgroups and the outgroup formed a taxon-quartet (Additional File 1). The majority of quartets was congruent with quartet topology A, from which our best ML tree can be derived (Additional File 1; Additional file 2-Table S12). Although we found evidence for confounding signal, this could not fully explain the quartet support. Thus, we consider that in this case genuine phylogenetic signal outweighs any confounding signal. Only when Chilopoda were excluded, the proportion of quartets supporting the quartet topology with Diplopoda+Symphyla and Pauropoda+Outgroup (Fig. 1, quartet topology C) gained considerable support. Quartet topology C, however, can be fully explained by confounding signal from non-randomly distributed data (compare permutation I and II, Additional file 2-Table S12). This quartet topology has never been obtained, neither by analyses of molecular nor of morphological data (Fig. 1 quartet topology C). AU tests rejected all trees derived from quartet topology B and quartet topology C (Fig. 4a). Our best ML tree (Fig. 2) was never rejected.

The second data set, STRICTaa_Pan (Additional File 1 and Additional File 2-Table S10), included all sequences of myriapods and pancrustaceans, while sequence data of chelicerates and onychophorans were excluded. ML tree inference resulted in a sister group relationship of Chilopoda and Diplopoda, with Symphyla as sister to this clade (Fig. 4b), the latter albeit with negligible support. In FcLM analyses of all four subsets (Additional File 1), the majority of quartets supported Chilopoda+Diplopoda, and confounding signal could never fully explain the results (Additional File 1 and Additional File 2-Table S13). This is again congruent with our remaining findings (Figs. 2 and 3). When either Chilopoda or Diplopoda were excluded, the majority of all drawn quartets in the FcLM analysis supported Pauropoda+Pancrustacea (Additional File 2-Table S13). The latter is incompatible with both, quartet topology A supported by the majority of drawn quartets, and quartet topology B supported by morphological evidence. FcLM permutations showed that this result cannot be fully explained by confounding signal. All AU tests on the data set including all myriapod subgroups and Pancrustacea but excluding Chelicerata and Onychophora rejected all trees which are not derived from quartet topology A (Fig. 4b).

**Fig. 5:**
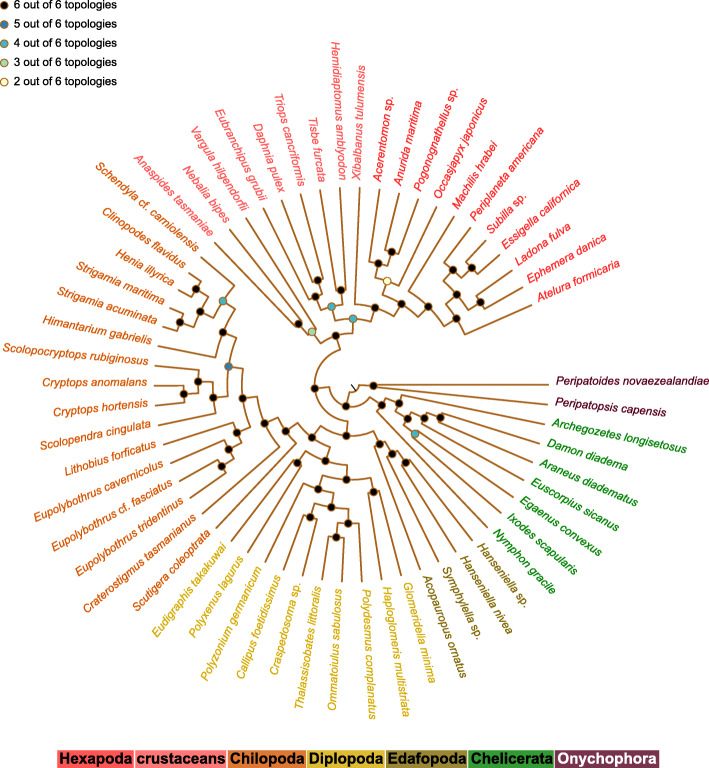
Summary of inferred ML topologies across all datasets. Circles indicate how often the split was found across the six tree topologies (Fig. 2 and Supplementary Figs. S7, S8, S9,S10,S11,S12, S13, S14, S15, S16 and S17). 50 out of 57 splits agree across all six ML topologies. Within myriapods, we found only two splits differing within Chilopoda

In summary, all trees but one, irrespective of the outgroup choice, are derivatives of our best supported quartet topology with Chilopoda+Diplopoda and Pauropoda+Symphyla (Fig. 1). Most of the splits correspond among all resulting topologies found in our study (Fig. 5). Only two splits within Myriapoda were not present in all topologies, both pertaining to internal relationships of Chilopoda. Most importantly, we found no support for a clade Diplopoda+Pauropoda (Dignatha), as present in morphological phylogenies.

## Discussion

While monophyletic Myriapoda, as well as their placement as sister group to Pancrustacea within Mandibulata is consistent with most recent studies (for a review, see [29]), our results regarding relationships among the four main subgroups are in conflict with the tree proposed by Fernandez and colleagues [2] and morphological evidence (for a review, see [3]). This is true for the placement of the internal root and regarding the underlying quartet topology (Fig. 1).

Chilopoda+Diplopoda and Pauropoda+Symphyla was the quartet topology that received the most support in all our analyses. Since rooting is possible at every branch, this quartet topology is congruent with five out of 15 possible trees (Fig. 1: first column). Most published phylogenies based on molecular data are derivatives of our best supported quartet topology [19–22]. However, the trees proposed by Rehm and colleagues [18] and Fernandez and colleagues [2] are derivatives of a quartet topology for which no support could be found in any of our analyses.

Fernandez and colleagues [2] hypothesised that their pauropod representative had been attracted towards equally long-branched pancrustacean lineages. In no tree inferred from our data sets the pauropod lineage showed a long branch. However, our pauropod representative clustered with Pancrustacea in FcLM when Chelicerata and Onychophora were excluded from the STRICT data set. We consider this result to be an artefact since the quartet topology is incongruent with all other analyses.

In none of our analyses did we find any support for the clade composed of Pauropoda and Diplopoda which was suggested by morphologists [3]. Instead, the majority of our analyses support a sister group relationship of Pauropoda and Symphyla. A sister group relationship of Chilopoda and Diplopoda, however not unambiguously supported, also seems likely. Our results strongly indicate that all remaining alternative trees are derivatives of one single quartet topology (quartet topology A, Fig. 1) which received the highest support.

Fernandez and colleagues [2] argue that the CAT model as implemented in PhyloBayes [30] outperforms partitioned approaches that assume SRH conditions in overcoming potential misleading effects due to heterogeneity among sites and lineages in data matrices [31, 32]. While this issue is still under debate (e.g. [33]), our data set, when applying a CAT-like mixture model with posterior mean site frequencies [25, 26] still favoured a sister group relationship of Pauropoda+Symphyla and not Diplopoda+Pauropoda. This result again was mirrored in AU tests. In addition, it is noteworthy that the CAT model does not account for among-lineage heterogeneity (Blanquart and Lartillot, pers. comm.) which is present in our and Fernandez [2] data sets (Additional File 3-Figs. S1 and S6). In addition, our quartet analyses including permutation approaches indicate that a quartet topology Diplopoda+Pauropoda may be biased by misleading signal derived from among-lineage heterogeneity and non-randomly distributed data (Fig. 3 and Additional File 2-Table S11). Quartet approaches such as FcLM or other quartet sampling methods have been suggested to complement tree inference with the aim to unmask alternative and confounding signal (e.g., [10, 34–36]).

While our tree conflicts with the distribution of morphological character states that support Dignatha, concerning Progoneata changing character polarisations is sufficient to avoid conflicts. A few morphological characters can be mentioned which are more consistent with our tree than with the traditional morphological tree. Apart of a series of comb lamellae on the mandibles [37], leg podomeres and trichobothria (bothriotricha) are very promising candidates for urgently needed comparative morphological and developmental studies among myriapods (see Additional File 1 for a more extensive discussion on morphology).

## Conclusions

Relationships among the four major myriapod subgroups remain among the most challenging splits in the arthropod tree. Our results based on phylogenomic data strongly contradict phylogenetic relationships among Chilopoda, Diplopoda, Pauropoda and Symphyla proposed by Fernandez and colleagues [2]. AU tests and quartet computation approaches could narrow down the space of possible trees to derivatives of a single quartet topology, in which Pauropoda+Symphyla oppose Chilopoda+Diplopoda. For this quartet topology we can rule out confounding signal such as among-lineage heterogeneity and non-randomly distributed data. We consider applied tests as useful complements of phylogenetic inference to discriminate topological conflicts from incongruencies due to differential internal rooting of the same quartet topology and to rule out confounding signal that might affect phylogenetic trees.

## Methods

We combined our own transcriptome data with public transcriptomic sequence data (or official gene sets) in a data set comprising 30 myriapod species, 27 species of the remaining arthropod groups, plus two onychophorans as outgroup species. From these 59 species in total, 42 were sequenced and de novo assembled for this study. A newly compiled ortholog set of 2716 single-copy and protein-encoding genes (ortholog groups, OGs) based on the OrthoDB v8 database (http://cegg.unige.ch/orthodb8) [38] was utilised to infer transcript orthology with Orthograph v. 0.5.6 [39]. Alignment, alignment refinement, removal of outlier sequences, identification and removal of ambiguously aligned sections, information content of gene partitions [40] and the compilation of optimised data matrices followed the procedures published by the 1KITE consortium (Supplements of e.g. [10, 15, 16]). Following the rationale of Dell’Ampio and colleagues [41] we compiled two concatenated main data sets with either maximal (STRICT) or high (RELAXED) coverage of included gene-partitions per species. The best partition schemes and best-fitting substitution models were estimated with PartitionFinder 2.0.0 [42] using a selection of models implemented in RAxML v8.2.4 [43] including one model that accounts for FreeRate heterogeneity [44]. Phylogenetic trees were calculated under the maximum likelihood optimality criterion using IQ-TREE (v1.4.2 and v.1.6.beta4) [45, 46] with a partitioned approach and additionally with an unpartitioned approach using a CAT-like protein mixture model [25, 26]. To summarise the support for the topology presented in Fig. 2, the trees from Supplementary Figs. S7, S8, S9,S10,S11,S12, S13, S14, S15, S16 and S17, were compared and visualised (Fig. 5) using the Newick Utilities tool [47]. To test competing hypotheses, we applied Four-cluster Likelihood-Mapping (FcLM) [10, 14] and the approximate unbiased test (AU-Test) [13] as implemented in IQ-TREE v.1.6.9. To finally identify possible confounding signal, FcLM permutation approaches were applied as introduced in previous phylogenomic studies [10, 15, 16]. To further test the inferred relationships of myriapod subgroups for a possible outgroup dependence, the two main data sets were modified including either only chelicerates and onychophorans as outgroup or only pancrustaceans as outgroup. These again were analysed by ML tree inference, AU tests and FcLM. All details on collecting data, sequencing, assembly, all procedures prior to phylogenetic analyses, settings and on applied tests are provided in Additional File 1 (Supplementary Text), Additional File 2 (Supplementary Tables) and Additional File 3 (Supplementary Figures). Raw and assembled transcriptome data are available at NCBI through the respective accession numbers (see Additional File 2-Table S1) and under the Umbrella BioProject accession PRJNA183205 (“The 1KITE project: evolution of insects”). Assemblies of previously published transcriptome data used for this study as well as other Supplementary data, e.g. the ortholog set, are available as Supplementary Archives on the DRYAD digital repository available with this study.

## Supplementary information

**Additional file 1: Supplementary Text.** Specifications on methods, with (i) Taxon sampling and tissue preservation, (ii) Library construction and de novo transcriptome sequencing, (iii) De novo assembly of transcriptome raw reads, (iv) Identification of single copy orthologs, (v) Multiple sequence alignment, refinement and removal of ambiguously aligned sections, (vi) Design of optimised data sets, (vii) Optimizing partition schemes, (viii) Phylogenetic tree inference and identification of rogue taxa, (ix) Tree testing: Alternative trees, confounding signal, and outgroup dependence of results, and (x) Composition of amino acid and nucleotide frequencies. Added is a section (xi) Morphological discussion.

**Additional file 2: Table S1.** Taxon sampling and accession numbers of raw and assembled transcriptome data of species included in this study. **Table S2.** Collection information. **Table S3.** Assembly statistics of published transcriptome data de novo assembled. **Table S4.** Contamination and assembly statistics of de novo assembled transcriptome data newly sequenced for this study. **Table S5.** Information and source of the reference species included in the ortholog set. **Table S6.** Orthograph statistics. **Table S7.** Group definitions to compile the data sets RELAXEDaa and RELAXEDnt (2nd codon positions). **Table S8.** Supermatrix diagnostics of final data sets compared with those analysed by Fernandez et al., 2018. **Table S9.** Overview of statistical bootstrap and transfer bootstrap support of selected clades. **Table S10.** Group definitions used for Four-cluster Likelihood Mapping (FcLM) analyses. **Table S11.** FcLM results testing the position of Myriapoda within Euarthropoda (Mandibulata versus Paradoxopoda). **Table S12.** Outgroup dependence: FcLM results testing myriapod relationships with Chelicerata and Onychophora as outgroup (data set STRICTaa_ChO). **Table S13.** Outgroup dependence: FcLM results testing myriapod relationships with Pancrustacea as outgroup (data set STRICTaa_Pan). **Table S14.** Amino acid and nucleotide frequencies of included species in data sets STRICTaa and STRICTnt.

**Additional file 3: Fig. S1.** Heat maps calculated with SymTest applying the Bowker‘s test on data sets STRICT and RELAXED. The heatmaps show the results of pairwise Bowker’s test as implemented in SymTest 2.0.47 analysing the supermatrices STRICT and RELAXED. The percentage of pairwise *p*-values < 0.05 rejecting SRH conditions are given in parentheses. Data set STRICT: a) amino acids (p-values < 0.05: 88.43%), b) 1st codon positions (p-values < 0.05: 99.3%), c) 2nd codon positions (p-values < 0.05: 85.15%), d) 3rd codon positions (p-values < 0.05: 100%). Data set RELAXED: e) amino acids (*p*-values < 0.05: 99.3%), f) 1st codon positions (p-values < 0.05: 99.94%), g) 2nd codon positions (p-values < 0.05: 96.9%), h) 3rd codon positions (p-values < 0.05: 100%). **Fig. S2.** Heat maps visualising the information content (IC) of our final data sets STRICTaa and RELAXEDaa calculated with Mare. The IC is color-coded in shades of blue, with darker shades representing higher IC and white squares indicate missing data, red squares (here not present) indicate meta-partitions with an IC = 0. a) data set STRICTaa. The 59 species are displayed in rows (x-axis) and the 215 meta-partitions (overall multiple sequence alignment length 95,797 amino acid sites) are shown in columns (y-axis). Overall information content: 0.303, matrix coverage in terms of meta-partitions: 100%. b) data set RELAXEDaa. The 59 species are displayed in rows (x-axis) and the 692 meta-partitions (overall multiple sequence alignment length 348,917 amino acid sites) are shown in columns (y-axis). Overall information content: 0.265, matrix coverage in terms of meta-partitions: 96.8%. Further diagnostics see Table S8. **Fig. S3.** Superalignment diagnostics of the data sets STRICTaa and RELAXEDaa. Heat maps indicating species-pairwise amino acid site-coverage inferred with AliStat of the sequences of 59 species. Low shared site-coverage are in shades of red and high shared site-coverage in shades of green. a) data set STRICTaa: Completeness alignment score (Ca): 82.53%, Maximum C-score for individual sequences (Cr_max): 97.04%, Minimum C-score for individual sequences (Cr_min): 39.41%. b) data set RELAXEDaa: Ca: 72.13%, Cr_max: 95.89%, Cr_min: 32.33%. Further diagnostics in Table S8. **Fig. S4.** Heat map visualising the information content (IC) of matrix 1 of Fernandez et al. (2018) calculated with Mare. The IC is color-coded in shades of blue, with darker shades representing higher IC and white squares indicate missing data. Red squares indicate gene partitions with an IC = 0. The 20 species are displayed in rows (x-axis) and the 229 gene partitions (overall multiple sequence alignment length 49,576 amino acid sites) are shown in columns (y-axis). Overall information content: 0.197, matrix coverage in terms of gene partitions: 78%. Further diagnostics, see Table S8. **Fig. S5.** Superalignment diagnostics of matrix 1 (Fernandez et al., 2018). The heat map indicates species-pairwise amino-acid site coverage of matrix 1 (20 species, Fernandez et al., 2018) inferred with AliStat. Low shared site-coverage are in shades of red and high shared site- coverage are in shades of green. Completeness alignment score (Ca): 72.67%, Maximum C-score for individual sequences (Cr_max): 97.08%, Minimum C-score for individual sequences (Cr_min): 10.19%. Further diagnostics in Table S8. **Fig. S6.** Heat map calculated with SymTest applying the Bowker‘s test on matrix 1 (Fernandez et al., 2018). The heatmap shows the results of pairwise Bowker’s test as implemented in SymTest 2.0.47 analysing matrix 1 (amino acid level) of Fernandez et al. (2018). Percentage of pairwise p-values < 0.05 rejecting SRH conditions: 64.74%. **Fig. S7.** Best ML tree inferred from the data set STRICTaa with transfer bootstrap support. The ML tree is identical with the ML tree displayed in Fig. 2a with statistical transfer bootstrap support (TBE) inferred from all bootstrap trees with Booster v. 0.1.2. Values range from 0 to 1 (rounded to two decimal places). The tree was rooted with Onychophora. **Fig. S8.** Inferred ML tree from the data set STRICTaa with the CAT-like mixture model + PSMF. Inferred ML tree from the data set STRICTaa using the unpartitioned approach applying the CAT-like mixture model + PSMF with statistical non-parametric bootstrap support inferred from 100 replicates. The tree was rooted with Onychophora. **Fig. S9.** Inferred ML tree from the data set STRICTaa with theCAT-like mixture model + PSMF with transfer bootstrap support. The ML tree is identical to the ML tree displayed in Fig. S8 with statistical transfer bootstrap support (TBE) inferred from all bootstrap trees with Booster v. 0.1.2. Values range from 0 to 1 (rounded to two decimal places). The tree was rooted with Onychophora. **Fig. S10.** Best ML tree inferred from the data set RELAXEDaa. Statistical non-parametric bootstrap support was inferred from 100 replicates. The tree was rooted with Onychophora. **Fig. S11.** Best ML tree inferred from the data set RELAXEDaa with transfer bootstrap support. The ML tree is identical to the ML tree displayed in Fig. S10 with statistical transfer bootstrap support (TBE) inferred from all bootstrap trees with Booster v. 0.1.2. Values range from 0 to 1 (rounded to two decimal places). The tree was rooted with Onychophora. **Fig. S12.** Inferred ML tree from the data set RELAXEDaa with the CAT-like mixture model + PSMF. Inferred ML tree from the data set RELAXEDaa using the unpartitioned approach applying the CAT-like mixture model + PSMF with statistical non-parametric bootstrap support inferred from 100 replicates. The tree was rooted with Onychophora. **Fig. S13.** Inferred ML tree from the data set RELAXEDaa with the CAT-like mixture model + PSMF with transfer bootstrap support. The ML tree is identical to the ML tree displayed in Fig. S12 with statistical transfer bootstrap support (TBE) inferred from all bootstrap trees with Booster v. 0.1.2. Values range from 0 to 1 (rounded to two decimal places). The tree was rooted with Onychophora. **Fig. S14.** Best ML tree inferred from the data set STRICTnt. Data set STRICTnt only includes 2nd codon positions. Statistical non-parametric bootstrap support was inferred from 100 replicates. The tree was rooted with Onychophora. **Fig. S15.** Best ML tree inferred from the data set STRICTnt with transfer bootstrap support. The ML tree is identical to the ML tree displayed in Fig. S14 with statistical transfer bootstrap support (TBE) inferred from all bootstrap trees with Booster v. 0.1.2. Values range from 0 to 1 (rounded to two decimal places). The tree was rooted with Onychophora. **Fig. S16.** Best ML tree inferred from the data set RELAXEDnt with non-parametric statistical bootstrap support. Data set RELAXEDnt only includes 2nd codon positions. Statistical non-parametric bootstrap support was inferred from 100 replicates. The tree was rooted with Onychophora. **Fig. S17.** Best ML tree inferred from the data set RELAXEDnt with transfer bootstrap support. The ML tree is identical to the ML tree displayed in **Fig. S16** with statistical transfer bootstrap support (TBE) inferred from all bootstrap trees with Booster v. 0.1.2. Values range from 0 to 1 (rounded to two decimal places). The tree was rooted with Onychophora. **Fig. S18.** Best ML tree inferred from the data set STRICTaa_ChO. Data set STRICTaa_ChO includes only Chelicerata and Onychophora as outgroup (excluding Pancrustacea). Statistical non-parametric bootstrap support was inferred from 100 replicates. The tree was rooted with Onychophora. **Fig. S19.** Best ML tree inferred from the data set STRICTaa_Pan. Data set STRICTaa_Pan includes only Pancrustacea as outgroup (excluding Chelicerata and Onychophora). Statistical non-parametric bootstrap support was inferred from 100 replicates. The tree was rooted with Pancrustacea.

## Data Availability

The datasets and additional information supporting the conclusions of this article are available in the Dryad repository “Data from: Four myriapod relatives – but who are sisters? No end to debates on relationships among the four major myriapod subgroups” (doi:10.5061/dryad.cvdncjt2r). DNA sequence data generated and analysed in this manuscript are deposited in a public database, NCBI. Accession numbers can be found in Table 1.

## Abbreviations

AU test: Approximately unbiased test
FcLM: Four-cluster Likelihood-Mapping
IC: Information content as calculated by the MARE software
ML: Maximum-Likelihood
OGS: Official gene set
SRA: NCBI Sequence Read Archive
RNA: Ribo nucleic acid
cDNA: Copy-DNA
DNA: Deoxyribonucleic acid
TSA: Transcriptome Shotgun Assembly
PCR: Polymerase Chain Reaction
PE: Paired end
bp: Base positions
AICc: Corrected Akaike information criterion
PMSF: Posterior mean site frequency profile
SRH: Global stationary, (time-) reversible and homogeneous conditions
MSA: Multiple sequence alignment
