# Supplementary material for: Four myriapod relatives – but who are sisters? No end to debates on relationships among the four major myriapod subgroups
Source: BMC Evol Biol. 2020 Nov 4;20:144. doi: 10.1186/s12862-020-01699-0 (PMC7640414; doi:10.1186/s12862-020-01699-0)
Supplement: Supplementary file 1 — Additional file 1: Supplementary Text. Specifications on methods, with (i) Taxon sampling and tissue preservation, (ii) Library construction and de novo transcriptome sequencing, (iii) De novo assembly of transcriptome raw reads, (iv) Identification of single copy orthologs, (v) Multiple sequence alignment, refinement and removal of ambiguously aligned sections, (vi) Design of optimised data sets, (vii) Optimizing partition schemes, (viii) Phylogenetic tree inference and identification of rogue taxa, (ix) Tree testing: Alternative trees, confounding signal, and outgroup dependence of results, and (x) Composition of amino acid and nucleotide frequencies. Added is a section (xi) Morphological discussion. [file 12862_2020_1699_MOESM1_ESM.doc]

**Four myriapod relatives – but who are sisters? No end to debates on relationships among the four major myriapod subgroups**

RUNNING HEAD: MYRIAPOD PHYLOGENOMICS

Nikolaus U. Szucsich, Daniela Bartel, Alexander Blanke, Alexander Böhm, Alexander Donath, Makiko Fukui, Simon Grove, Shanlin Liu, Oliver Macek, Ryuichiro Machida, Bernhard Misof, Yasutaka Nakagaki, Lars Podsiadlowski, Kaoru Sekiya, Shigekazu Tomizuka, Björn von M. Reumont, Robert M. Waterhouse, Manfred Walzl, Guanliang Meng, Xin Zhou, Günther Pass, Karen Meusemann

**Additional File 1: Supplementary Text**

### Note that in several files (FASTA, NEXUS, NEWICK) sample names are slightly different. They refer to following species:

| **Name in files** | **valid species name** | **Note** |
| --- | --- | --- |
| Peripatopsis_capensis_F2014 | *Peripatopsis capensis* | from Fernandez et al., 2014, this species is listed in NCBI under *Peripatopsis overbergiensis* (PRJNA236598) |
| Schendyla_cf_carniolensis | *Schendyla carniolensis* |  |
| Cryptops_hortensis_F2014 | *Cryptops hortensis* | from Fernandez et al., 2014 |
| Eupolybothrus_cavernicolus_sp | *Eupolybothrus cavernicolus* |  |
| Eupolybothrus_cf_fasciatus | *Eupolybothrus fasciatus* |  |
| Acerentomon_maius | *Acerentomon* sp. | corrected to sp. Since not every single specimen could be identified as *A. maius* |
| Occasjapyx_japonicus_neu | *Occasjapyx japonicus* | assembly released with this study, (Table S1, PRJNA286654) |
| EDANI | *Ephemera danica* | official gene set |
| LFUL | *Ladona fulva* | official gene set |
| Xanthostigma_xanthostigma | *Subilla* sp. | identification to *Subilla* sp. corrected in 2018 |

### Taxon sampling and tissue preservation

We collected 42 species in total: 6 chelicerates, 8 crustaceans, 27 myriapods (including 13 chilopods, 10 diplopods, 3 symphylans and one pauropod), plus one additional onychophoran for transcriptome sequencing, BioProject Accession numbers are provided in Additional File 2-Table S1. Collected individuals were narcotised with CO_2_ and either directly smashed in RNAlater^TM^ or preserved in liquid nitrogen and subsequently ground in RNAlater^TM^. Information about preservation method, number of individuals, sex, developmental stages, and collection localities are given in Additional File 2-Table S2. Our taxon sampling was complemented by transcriptome data of twelve published species. Additionally, four species were retrieved from NCBI Sequence Read Archive (SRA) and via official gene sets (OGS). We only included taxa in our further analyses with a minimum of 50% coverage of the ortholog set (see below).

The final taxon sampling comprised thirty myriapod species (16 chilopods, ten diplopods, three symphylans and one pauropod) and 29 outgroup species (two onychophorans, seven chelicerates, nine crustaceans and eleven hexapods).

### Library construction and de novo transcriptome sequencing

Tissue samples preserved in RNAlater^TM^ were sent to the BGI-Shenzhen, China. Messenger RNA (mRNA) was isolated using the Dynabeads mRNA Purification Kit (Invitrogen, Grand Island, NY, USA) and subsequently sheared using RNA fragmentation reagent (Ambion, Austin, Texas, US) at 72°C. The cleaved RNA fragments were transcribed into first-strand cDNA using SuperScript™II Reverse Transcriptase (Invitrogen, Grand Island, NY, USA) and random N6 primer (IDT). The second-strand cDNA was synthesised using RNase H (Invitrogen, Grand Island, NY, USA) and DNA polymerase I (New England BioLabs, Ipswich, MA, USA). The double-stranded cDNA was processed in an in-house pipeline including end-repair, a single ‘A’ base addition, adapter ligation, and size selection on agarose gels (250 ± 20 bp). The product was then indexed and PCR amplified to finalise library preparation for the paired-end cDNA.

For species with a total RNA amount of < 0.8 µg and/or < 4 µg, and an additional species (*Glomeridella minima)* with a high amount of 5S rRNA (due to ribosomal contaminations), libraries were constructed using TruSeq mRNA Library Prep Kit (Illumina, Ca. USA). Purified mRNA was sheared into length of 160 - 170 bp using divalent cations at 98°C, while skipping the gel based size selection and purification step. Verification of the cDNA fragment size and concentration was accomplished using an Agilent 2100 Bioanalyzer and an ABI StepOnePlus Real-Time PCR machine.

The cDNA libraries were subsequently sequenced on an Illumina HiSeq2000 with strategies of 150 bp paired-ends (PE) and 90 PE, respectively, for standard and TruSeq libraries, generating ca. 2.5Gbases raw data for each specimen. Details on RNA extraction, cDNA library preparation and HiSeq Illumina sequencing is similar as described in Misof and colleagues [1], as well as Peters and colleagues [2].

### De novo assembly of transcriptome raw reads

We used two different assembly strategies:

1) Published short read data of selected species from Stoev and colleagues and from Fernandez and colleagues ([3, 4] and Additional File 2-Table S1) were downloaded from NCBI SRA and assembled with *Trinity v2.1.1* [5, 6] using quality trimming by the integrated *Trimmomatic* with modified trimming parameters (SLIDINGWINDOW:4:30 LEADING:30 TRAILING:30 MINLEN:50). Assembly statistics are summarised in Additional File 2-Table S3.

2) Raw reads from species novel sequenced in the present study (Umbrella project 1KITE with two subprojects, „VIEART“ and „1KITE”, Additional File 2-Table S1) were assembled using the assembler *SOAPdenovo-Trans-31kmer* ([7], version 1.02). Raw data were pre-processed discarding (i) reads with adapter contamination (minimum length of the alignment: 15 bp; at most 3 mismatches), (ii) reads that included > 10 Ns, and (iii) reads that included > 50 base pairs of low quality (*i.e.*, Phred quality score: 2, ASCII 66 "B", Illumina 1.5+ Phred+64). All remaining reads were used for *de novo* assembly. For details on the assembly process, see [7]. We slightly modified the default settings of [7] in the contig forming step: linear k-mers, *i.e.* k-mers with a single out-degree, were merged to form edges and different edges were linked by arcs. Arcs with an abundance < 5% of the total out-degrees or < 2% of the total in-degrees were excluded. Subsequently, edges with an average abundance ≥ 3 were reported as contigs.

We searched the *de novo* assembled transcriptomes for cross-library contamination which can occur when pooling single index-tagged NGS libraries on the same Illumina NGS sequencer lane, using the search strategy outlined by Mayer and colleagues [8]. We compared each transcriptome assembly with all other assemblies sequenced within 1KITE in all batches (1KITE samples) or respectively sequenced in the same batch (VIEART samples) using BLASTN applying Megablast of the BLAST+ program suite (version 2.2.31, [9], settings: blastn -task megablast, -evalue 1e-5, -outfmt 6 -out megablast.out). In case the BLASTN search identified transcripts that shared over a length of at least 200 bp a nucleotide sequence identity of at least 98% and E-value <= 1e-5, we used the coverage depth to determine which transcript was likely the original sequence (by being more abundant) and which one likely represented the contamination (by being less abundant). We used as coverage depth of a given transcript the average k-mer coverage statistic provided by the assembly software *SOAPdenovo-Trans-31kmer* [7]. Having identified transcripts sharing a high sequence identity, we applied the following procedures that slightly differed between samples of the two subprojects („VIEART“ and „1KITE“) because of organisational reasons.

VIEART samples: (i) If two transcripts of two different samples differed more than 10-fold, we removed the transcript with the lower relative coverage from the corresponding assembly; (ii) If the coverage of the two transcripts in question differed less than 10 fold, then we conservatively removed both of them from the two corresponding assemblies.

1KITE samples: (i) If two transcripts differed more than 2-fold in their relative coverage, we removed the transcript with the lower relative coverage from the corresponding assembly; (ii) If the coverage of the two transcripts in question differed 2-fold or less, we conservatively removed both of them from the two corresponding assemblies. This procedure helped to remove putative foreign contaminations (e.g., from third party libraries sequenced on the same lane, but not present in our analyses). It also meant that in case of multiple highly similar sequences, we retained only the single transcript with the highest relative coverage (see above). Details are described e.g., in Peters and colleagues [2].

Along with data deposition at the NCBI Transcriptome Shotgun Assembly (TSA), assemblies were searched for vector and linker/adapter sequences using *VecScreen* (http://www.ncbi.nlm.nih.gov/tools/vecscreen/) and the *UniVec* database version 9.0 (http://www.ncbi.nlm.nih.gov/tools/vecscreen/univec). We removed both terminal and internal hits. The removal of internal hits resulted in a split of the respective contigs/scaffolds in two parts. Furthermore, we removed transcripts from the assembled transcriptomes that NCBI identified as possibly foreign contaminations (see below).

We removed per assembled transcript library between 2,064 (0.01% of the unfiltered assembled transcriptome) and 43,199 (0.13% of the unfiltered assembled transcriptome) nucleotides, as they likely or possibly represented contamination, detailed information is summarised in Additional File 2-Table S4. Data (raw reads and cleaned assemblies) are available at NCBI SRA and TSA archives through the Umbrella BioProject accession PRJNA183205 "The 1KITE project: evolution of insects" and the respective BioProject accession numbers (Additional File 2-Table S1), *de novo* assemblies from other than 1KITE published data are provided as Supplementary Archive 1.

### Identification of single copy orthologs

We compiled a reference set of 2,716 ortholog groups (OGs, *i.e.* single-copy, nuclear protein-coding genes) using the official gene sets (OGS) available from selected published genomes *Ixodes scapularis*, *Daphnia pulex*, *Strigamia* *maritima*, and *Zootermopsis* *nevadensis* utilising the *OrthoDB* database version 8 (http://cegg.unige.ch/orthodb8) [10]. The hierarchical split was set to Arthropoda. For selected species, the search was restricted to single-copy (copies = 1), for others, the option was set to “unknown number of copies”. For the reciprocal search of candidate transcripts against the full OGS, we downloaded the OGS on amino acid level and respective coding nucleotide sequences (cds) of the four selected reference species. We retained only the isoform, which *OrthoDB* v8 determined as orthologous. We removed terminal stop codons in the protein files and used the script “make-ogs-corresponding.pl” to search and correct for inconsistencies between the amino acid sequences and corresponding nucleotide sequences of each OGS (information of used OGS is provided in Additional File 2-Table S5). The list of OGs and the OGS on protein and cds level (Supplementary Archive S2) served as input for the assignment of our transcripts to single-copy orthologs. We used *Orthograph* v. 0.5.6 [11] to search all transcripts against our ortholog reference set. We applied relaxed reciprocal search against any of the reference species and the best reciprocal hit (BRH) criterion was fulfilled if the first reciprocal hit was a protein sequence that was also part of the profile Hidden Markov Model (pHMM), irrespective of the species. We also allowed for a frame shift-corrected transcriptional extension of each transcript beyond the region of the transcript for which the BRH criterion was fulfilled (option: extend-orf = 1, orf-overlap-minimum = 0.5). Selenocysteine (“U“) was replaced with X (respectively with “NNN“ on nucleotide level) to avoid problems in downstream analyses. Other settings were left to default. Sequence headers were subsequently reformatted (to *HaMStR-ad* format, see *Orthograph* manual) and results were summarised for all taxa with accompanying helper scripts provided with the *Orthograph* package. Thereby, terminal stop codons were removed and internal stop codons replaced with X or NNN in all amino acid and corresponding nucleotide sequences, respectively.

On average, transcripts of 54 assembled libraries and two official gene sets (*i.e.* *Ephemera danica* and *Ladona fulva* not included in our ortholog set) could be assigned to one of the 2,716 target genes (82.86% if the ortholog set) ranging from 1,382 OGs (*Vargula hilgendorfii*) to maximally 2,625 OGs (*Clinopodes flavidus*). A detailed summary is provided in Additional File 2-Table S6.

#### *Multiple sequence alignment, refinement and removal of ambiguously aligned sections*

We generated a multiple sequence alignment (MSA) on amino acid level of each OGs using *MAFFT* [12] v7.221 with the L-INS-i algorithm. We subsequently checked each amino acid MSA for putative outlier sequences and identified 1,246 putative outliers in altogether 606 amino acid MSAs. Alignment refinement of putative outliers, a second check and subsequent removal of final outlier sequences were performed as explained in the Supplementary material of Misof and colleagues [1]. The refinement step succeeded in correctly re-aligning 357 sequences in 128 amino acid MSAs. Remaining 889 outliers (in 478 MSAs) were discarded. Corresponding sequences on nucleotide level were removed accordingly. Moreover, all data of *Zootermopsis nevadensis* were removed since this species was excluded from subsequent analyses. Subsequently all sites with gaps only were discarded. Corresponding MSAs on the nucleotide level were generated using a modified version of the software *Pal2Nal* [13] version 14; see [1] for details on the modification) using the amino acid MSAs as blue-print.

We identified ambiguous or randomly aligned MSA sections of the amino acid MSAs with a modified version of *Aliscore* v1.2 [14, 2, 15]. We considered the maximum number of pairwise sequence comparisons for each MSA (option -r), and the -e option for gap-rich data sets, leaving remaining settings to defaults. Subsequently all MSA sections, which were indicated as randomly similar aligned, were removed with *Alicut* v2.3 (https://www.zfmk.de) from the amino acid and nucleotide MSAs, respectively. On average, 20.7% of each MSA was excluded. MSAs of 30 OGs remained unmasked since no ambiguously aligned sections had been identified. Subsequently MSAs were concatenated with *FasConCat* v1.0 [16] to a supermatrix on amino acid level (spanning a superalignment length of 1,051,684 amino acid sites) and a supermatrix on nucleotide level (3,155,052 nucleotide sites). Each composed of 2,716 gene-boundary-based partitions with 59 taxa.

### Design of optimised data sets

To optimise our data sets, the putative information content (IC) on amino acid level for each gene-partition was calculated using *MARE* [17], version 1.2-rc with default settings. Gene-partitions with an IC=0 were subsequently removed from both, the amino acid and the nucleotide supermatrix. Furthermore, we deleted all sites containing only gaps and/or missing data (X/NNN), which lead to supermatrices with 2,711 gene-partitions spanning an alignment length of 1,049,581 amino acid sites and3,148,743 nucleotide sites, respectively.

Following Dell’Ampio and [18] colleagues and Misof and colleagues [1], we used the above data to design the following data sets:

(i) a STRICT data set: we only kept gene partitions that were present in each of the 59 taxa, *i.e.* 100% coverage in terms of genes hereinafter called STRICTaa (amino acid) and STRICTnt (nucleotide) data set, respectively

(ii) the more relaxed data set: here, we kept all gene partitions for which at least one representative taxon of selected groups of interest were present, hereinafter called RELAXEDaa and RELAXEDnt data set, respectively. Group definitions are provided in Additional File 2-Table S7.

To explore putative among-lineage heterogeneity (see [1] for a rationale), we used *SymTest* version 2.0.44 (available from https://github.com/ottmi/symtest, [19] to evaluate, whether or not our data sets matched globally stationary, time-reversible and homogeneous (SRH) conditions [20, 21]. On the nucleotide level, we applied *SymTest* on the 1^st^, 2^nd^ and 3^rd^ codon positions separately. *SymTest* generates heatmaps based on p-values obtained from the implemented Bowker’s matched pairs test of symmetry [22] to determine which sequence pairs have evolved under globally SRH conditions. Keeping only the 2^nd^ codon position for both data sets lowered among-lineage heterogeneity for all further analyses on nucleotide level (Additional File 3-Fig. S1). A possible impact of violating the SRH assumptions during maximum likelihood (ML) tree inference was tested with Four-cluster Likelihood-Mapping (FcLM) including permutation approaches as outlined in previous studies ([1, 2, 23], see below).

The STRICT data sets included 292 gene partitions with 95,797 amino acid and 95,797 nucleotide sites (2^nd^ codon positions), respectively. Merging of small partitions (below 100 amino acid and 300 nucleotide sites) downsized the number of gene-partitions to 284. The RELAXED data sets included 988 gene-partitions (348,917 sites). Merging of small partitions downsized the number of gene-partitions to 953. These four data sets with the respective partition schemes served as input for partition merging.

*Optimising partition schemes*

For each of the four optimised data sets STRICTaa, STRICTnt, RELAXEDaa, RELAXEDnt, gene-partitions that can be analysed under the same substitution model were merged and the best fitting model substitution model was estimated for each merged partition with *PartitionFinder*2.0.0 [24], pre-release 11 using *RAxML* v8.2.4 [25]. We restricted amino acid substitution models to LG [26], WAG [27], DCMut [28], JTT [29], and BLOSUM62 [30]. A GAMMA (+G) distribution or +G with empirical base frequencies (+F) and the LG4X [31] accounting for FreeRate heterogeneity was assumed with four rate categories. For the nucleotide data sets, we used the substitution model GTR due to restrictions in *RAxML*, either with +G, with +I accounting for invariant sites or with G+I, respectively. The best partitioning scheme and the best fitting model for each meta-partition was selected based on the corrected *Akaike Information Criterion* (AICc) [32]. Further settings were: branchlengths linked, --raxml, --all-states, --min-subset-size 100 (for amino-acid data sets), --min-subset-size 300 (for the nucleotide data sets), --rcluster-max (twice the amount of initial number of gene partitions), --rcluster-percent 50, -q, --ml-tree. Data set STRICTaa finally included 215 meta-partitions (alignment length: 95,797 amino acid sites), the data set STRICTnt included 139 meta-partitions (alignment length: 287,391 nucleotide sites). Merging partitions for the data set RELAXEDaa resulted in 692 meta-partitions, (348,917 amino-acid sites); the best partition scheme for data set RELAXEDnt included 468 meta-partitions (95,797 nucleotide sites). We again calculated overall information content (IC) and meta-partition coverage of the amino acid supermatrices with *MARE* v1.2-rc. The overall completeness score for the alignment (Ca), the minimum C-score for individual sequences (Cr_min), a maximum C-score for individual sequences (Cr_max) were calculated using *AliStat* version 1.6 (https://github.com/thomaskf/AliStat) [33]. In addition, we generated corresponding heat maps of the distribution of missing data from pairwise sequence comparisons of the sequences in the supermatrix. Optimised data sets with the best partitions schemes and selected substitution models served as input for all subsequent phylogenetic analyses (Supplementary Archive S3).

Data matrix diagnostics (overall information content, coverage in terms of (meta-) partitions, and alignment completeness scores) of our data sets are provided in Additional File 2-Table S8 and Additional File 3-Figs. S2-S3, complemented with diagnostics of the three matrices analysed by Fernandez and colleagues [34] for comparison (see also Additional File 3-Figs. S4-S6).

*Phylogenetic tree inference and identification of rogue taxa*

Tree searches were performed for both STRICT and RELAXED data sets (aa and nt, respectively) with a maximum-likelihood (ML) approach using *IQ-TREE* (v1.4.2, v.1.6.beta4, and v.1.6.9) [35, 36]. For each data set, we performed 50 ML tree searches with randomised starting trees. Tree searches were performed using a partitioned approach with the best partition scheme, the selected models and the edge-proportional partition model (option -spp). Additional settings were --gmedian and --numstop 300. All other settings were left to default. Statistical support was derived from 100 non-parametric, slow bootstrap replicates and subsequently mapped onto the ML tree with the best log-likelihood score out of the 50 inferred ML trees. The number of unique tree topologies were checked with *Uniquetree* version 1.9, (Wong, available upon request). We ensured convergence of bootstrap replicates *a posteriori* with *RAxML* version 8.2.11 (options: -autoMRE -B 0.03 --bootstop-perms=10000) starting with random seeds [37] ten times independently. Bootstrap replicates converged for all STRICT and RELAXED data sets always after 50 replicates. Additionally, we inferred transfer bootstrap support for each split with the software *Booster* v. 0.1.2 [38]. Best ML trees with statistical support from non-parametric bootstraps and transfer bootstrap support for data sets STRICTaa, STRICTnt, RELAXEDaa, and RELAXEDnt are provided as Supplementary Archive S4.

We checked the STRICT and RELAXED data sets for rogue taxa using *RogueNaRok* version 1.0 [39] providing for each data set the best ML tree and otherwise default settings. All our data sets were found to be free from rogue taxa.

The CAT model as implemented in *PhyloBayes* [40], a non-parametric mixture model accounting for among-site heterogeneity has been suggested to be more appropriate for inferring phylogenetic trees than using a partitioned-based approach (e.g. [34, 41, 42], but see also [43]). The CAT model in *PhyloBayes* and the CAT-like C60 models, which assume a GAMMA distribution, do, however, only account for among-site, but not for among-lineage heterogeneity (Lartillot & Blanquart, personal communication 2018). The only derivation of *Phylobayes* (NH_PhyloBayes, [44]) that accounts for among-lineage heterogeneity (CAT-BP model) cannot cope with large data sets like ours (Blanquart 2017, pers. comm., see also [23]). We instead applied on our amino acid data sets the CAT-like profile C60 mixture model (see [45]), inferring one ML tree search on the STRICT and on the RELAXED data set, both unpartitioned, with the posterior mean site frequency (PMSF) approach [46]. Since the LG matrix (either LG+G or LG4X) was the most frequently selected substitution model in previous analyses, we choose the LG instead of the Poisson matrix (option LG+C60+G+F), the option –gmedian and used the best ML trees from previous partitioned analyses of the STRICTaa and RELAXEDaa analyses as guide trees. Statistical support was inferred from 100 non-parametric bootstrap replicates. Transfer statistical bootstrap support was again calculated with *Booster* v. 0.1.2.

We rooted all ML trees with onychophorans using *SeaView* (v.4.5.4) [47] and edited phylograms with *Inkscape* (v.0.91) ([www.inkscape.org](http://www.inkscape.org/)).

Comparing all inferred ML trees across the data sets STRICT, RELAXED, different data types (amino acid and nucleotide level) and tree inference approaches (partitioned vs. unpartitioned approach with the CAT-like mixture model), relationships were always identical. There was only one exception with a minor difference irrelevant for relationships among the four myriapod subgroups: the 50 ML trees inferred from the data set RELAXEDaa differed with respect to internal relationships of Geophilomorpha: Himantariidae and Schendylidae were either inferred as sister groups (with negligible support) or Himantariidae were inferred to a clade (Schendylidae + remaining geophilomorphan species), albeit weakly supported, but found in the majority and across all data sets.

In all best ML trees inferred from the STRICTaa, STRICTnt, RELAXEDaa, and RELAXEDnt data sets, irrespective of using the partitioned-based or the unpartitioned CAT-like C60 profile mixture model approach + PMSF, Myriapoda was placed as sister to Pancrustacea supporting Mandibulata with maximal support. The same holds for the monophyly of Myriapoda with maximal statistical bootstrap and transfer bootstrap support (Additional File 2-Table S9, Fig. 2, Additional File 3-Figs. S7-S17).

The four major myriapod lineages Symphyla, Pauropoda, Chilopoda, Diplopoda were always inferred as monophyletic with maximal statistical support in all four data sets (Additional File 2-Table S9, Fig. 2, Additional File 3-Figs. S7-S17), irrespective of data type (amino acid and nucleotide level) and approach (ML-partitioned, non-parametric and transfer bootstrap support and CAT-like mixture model unpartitioned). Phylogenetic relationships among the four major lineages were again similar in all data sets (Additional File 2-Table S9, Fig. 2, Additional File 3-Figs. S7-S17): Symphyla was always inferred as sister to the pauropod (with maximal or strong support). A sister group relationship of Chilopoda and Diplopoda was inferred either with strong (nucleotide data sets) or moderate support.

### Tree testing: Alternative trees, confounding signal, and outgroup dependence of results

To test for (i) signal for alternative trees, (ii) misleading confounding signal due to model violation like among-lineage heterogeneity and distribution of (missing) data, as well as (iii) the dependence of internal relationships among myriapod subgroups on outgroup choice, we performed (a) AU-tests and (b) Four-cluster Likelihood-Mapping analyses, and additionally (c) restricted our data sets to either Pancrustacea or Chelicerata+Onychophora as the sole outgroup.

***(a) AU-tests*:** Using the data sets STRICTaa and STRICTnt, five alternative phylogenetic hypotheses were tested with respect to the four major myriapod subgroups Symphyla, Pauropoda, Chilopoda and Diplopoda based upon published and our alternative trees against the best ML tree of our data set STRICTaa. We used the approximate unbiased (AU) test [48] as implemented in *IQ-TREE* v. 1.6.9 with 100,000 RELL replicates for each test respectively. Input tree topologies used in the AU test and when deviating from the best ML tree, were edited and manipulated using the software *Mesquite* v. 3.1 [49].

Hypotheses tested (see Fig. 2, 4):

(1) Hypothesis A1: our best ML tree (Fig. 2):

((Chilopoda, Diplopoda), (Symphyla, Pauropoda)) – monophyletic Edafopoda opposed by a clade uniting centipedes and millipedes

(2) Hypothesis A2: alternative tree which was inferred when the outgroup was restricted to Chelicerata and Onychophora only (see below), and tree suggested by Regier and colleagues [50] and Zwick and colleagues [51] as most likely:

(Chilopoda, (Diplopoda, (Symphyla, Pauropoda))) – monophyletic Edafopoda within Progoneata

(3) Hypothesis A3: alternative tree which was inferred when the outgroup was restricted to Pancrustacea only (see below):

(Pauropoda, (Symphyla, (Diplopoda, Chilopoda)))

(4) Hypothesis B1: tree suggested by Fernández and colleagues [34] as most likely and by studies using morphological traits (see [52]):

(Chilopoda, (Symphyla, (Diplopoda, Pauropoda))); note that we analysed two variations of this tree, once with Geophilomorpha and Scolopendromorpha being sister groups (like in our preferred tree), the second with Lithobiomorpha and Scolopendromorpha as sisters like previously inferred [34]. All AU tests were insensitive to this variation, thus they are merged in Fig. 2 and Fig. 4

(5) Hypothesis B2: alternative tree as suggested by morphological studies (note that Trignatha of Tiegs (1947) [53] included insects, with paraphyletic Myriapoda, therefore in all figures displayed as “Trignatha”):

((Chilopoda, Symphyla),(Diplopoda, Pauropoda))

Only trees derived from the quartet topology for which we found most support, namely Chilopoda+Diplopoda opposing Symphyla+Pauropoda passed the AU-test of the data set STRICT (Fig. 2).

***(b) Four-cluster Likelihood-Mapping analyses:*** Beyond alternative signal inherent in a data set which is not necessarily reflected in a phylogenetic tree, among-lineage heterogeneity and distribution of (missing) data - although the latter was minimised in our STRICT data sets - can lead to model violation during tree inference and to misleading/biased phylogenies. We tested our STRICTaa data set for signal for alternative relationships among Symphyla, Pauropoda, Chilopoda, and Diplopoda with Four-cluster Likelihood-Mapping [54]. To identify potential confounding signal that might affect phylogenetic ML tree inference, specifically, heterogeneity among lineages and violating SRH conditions and non-random distributed data, we additionally performed FcLM with three permuted data sets without phylogenetic signal (e.g., [1, 2, 23]). We then compared the results with the FcLM results from the original, non-permuted data. Summarised, in permutation I, all phylogenetic signal was destroyed but heterogeneity among lineages violating SRH conditions and non-random data distribution was left untouched. Permutation II left the non-random data distribution untouched, but homogeneity among lineages was guaranteed. Permutation III was similar to permutation II but the data distribution was randomised as well. FcLM was performed on original and permuted data sets using likelihood mapping as implemented in *IQ-TREE* (v.1.6.beta4).

For each hypothesis tested, we grouped species into four groups. Species included in each group, the number of drawn quartets and additional information are given in Additional File 2-Table S10.

With FcLM we assessed:

1. the position of Myriapoda within Euarthropoda (Chelicerata, Myriapoda, Pancrustacea, remaining species considered as root/outgroup) and
2. relationships among the myriapod subgroups Chilopoda, Diplopoda, Symphyla, Pauropoda. Refer to the main text and to Additional File 2-Table S11 for results and discussion of the relationships among Chilopoda, Diplopoda, Symphyla, Pauropoda.

***(c) outgroup dependence of results:*** To test for the impact of chosen outgroup on the results using the full taxon sampling, as previously supposed (Fernandez et al. 2018), we compiled two data subsets, both including all myriapod species with either (a) only chelicerates and onychophorans (STRICTaa_ChO) or (b) only Pancrustacea (STRICTaa_Pan) as outgroups.

From these data subsets, the best ML tree with statistical non-parametric bootstrap support was inferred with settings described earlier (50 ML tree searches, 100 bootstrap replicates, same settings). Both data sets resulted in different trees, both of them, however, derivatives of topology A (Fig. 1). Restricting outgroups to Chelicerata and Onychophora left Chilopoda as sister to all remaining Myriapoda (Fig. 4, Additional File 3-Fig. S18). Restriction to Pancrustacea as outgroup resulted in Pauropoda being sister to the remaining Myriapoda (Fig. 4, Additional File 3-Fig. S19).

Additionally, we tested on both data subsets whether or not the six alternative trees reflecting different hypotheses (see previous section) were rejected applying the AU test (Fig. 4).

Since FcLM analyses are restricted to four groups only (each terminal of a quartet represents one taxonomic group/assemblage), outgroup dependence could be tested only in subsets. Each data subset excluded one of the four myriapod subgroups. This was done for both data subsets, STRICTaa_ChO and STRICTaa_Pan (Additional File 2-Table S10), to check for possible influence on non-myriapod outgroup taxa when inferring phylogenetic relationships. Results of the FcLM are provided in Additional File 2-Table S12 for the data subset STRICTaa_ChO with only Chelicerata and Onychophora as outgroup and in Additional File 2-Table S13 for the data subset STRICTaa_Pan with only Pancrustacea as outgroup.

### Composition of amino acid and nucleotide frequencies

For the STRICTaa and STRICTnt data set, we calculated amino acid and nucleotide frequencies for each taxon (species) by custom-made Perl scripts. No clear pattern for a taxon-specific bias was observed within the data set (Additional File 2-Table S14).

### **Morphological discussion**

Our best tree is in conflict with Progoneata and Dignatha, two taxa erected on the basis of putative synapomorphies from morphological and developmental characters. While for Dignatha the proposed distribution of the character states is in conflict with our best tree, for Progoneata the distribution of the states itself is not problematic, but our best tree disagrees with traditional character polarisations.

Character polarisation is a tricky task, especially for deep splits where outgroup comparison often gives very few clues of which state might be seen as plesiomorphic. Sometimes scientists do not question “traditional” views on character polarisation, even if new evidence results in new views on the phylogeny. This also holds true at least for some characters in myriapods. The terminal position of the genital pore is usually assessed to be plesiomorphic, leaving the anterior position in Diplopoda, Symphyla and Pauropoda a possible synapomorphy for Progoneata. This character polarisation was more parsimonious when insects were suggested to be the closest relatives of myriapods. However, this is not well underpinned by the distribution of genital pores in Chelicerata or early crustacean splits within Pancrustacea. In most chelicerates, the genital pore is at the anterior part of the opisthosoma [55]. While homologisation of segments among arthropod major groups is at least debatable, the position quite well fits with the progoneate character state [56].

Character polarisation can likewise easily be questioned as to the missing palps mentioned as possible apomorphy supporting Progoneata.

The bothriotricha of Pauropoda and Symphyla match, both in (ultra-)structure and position. Among all Diplopoda, bothriotricha are present in Penicillata alone – both the (ultra-)structure and the position at the head differ from symphylans and pauropods, so that even homology is questionable.

Additionally, Progoneata was assessed to be a weakly supported taxon by many authors. This is different for Dignatha. Dohle [57] mentioned several correspondences between Pauropoda and Diplopoda, which he assessed to be well supported apomorphies: (i) the lack of appendages in the second maxillary segment, (ii) the formation of a 'lower lip' by the appendages of the first maxillary segment and the intervening sternite, (iii) genital pores at the base of the second trunk leg pair, (iv) sternal spiracles which open into a tracheal pouch giving rise to an apodeme and to tracheae, (v) a pupoid stage, and (vi) a first free-living juvenile with three pairs of legs.

Everyone has to confess that the mere number of mentioned synapomorphies sounds convincing, even if some of the characters can be found within Pauropoda only in the understudied Hexamerocerata and/ or within Diplopoda only in Penicillata. Remarkably, in Hexamerocerata, the first free-living juvenile has six legs [58], a number matching the 6-7 pairs of the first juveniles in Symphyla [59]. At least here, the plesiomorphic state within Pauropoda is ambiguous.

The sternal structures with tracheal pouch and apodeme are likewise controversial. In both, Pauropoda and Symphyla, coxal apodemes are described as thin chitinous ingrowths from the anterior face of each coxa, running caudad and mediad [53, 60]. Thus, homology of the coxal apodemes of Symphyla and Pauropoda is well corroborated by a corresponding origin at the anterior face of the coxa. By contrast, a homology to the tracheal pouches in Diplopoda is questionable, since the latter structure mostly does not originate at the coxa, but at the sternite.

The namesgiving dignathy still remains the strongest argument for Dignatha. Little can be added there, since our knowledge on the ontogenetic characters still depends on the work of Tiegs [53]. As to mouthparts, a study on Hexamerocerata would be highly desirable, since they have chitinized mouthparts, while Tetramerocerata (including *Pauropus* studied by Tiegs) have weakly sclerotised mouthparts which are used for sucking. The mouthparts of Pauropoda clearly differ from symphylan mouthparts. The shared terminology used in Diplopoda and Pauropoda, nevertheless should not be mixed with homology. A re-study of pauropod mouthparts is one of the biggest gaps preventing a better understanding of myriapod phylogeny.

A few morphological characters can be mentioned which are more consistent with our best tree than with the traditional tree with Progoneata and Dignatha.

In myriapods, the number of podomeres differs, ranging from six in Symphyla and Pauropoda, to seven in Chilopoda, and eight in Diplopoda [61, 62]. While again character polarisation seems nearly impossible, the shared character state of Symphyla and Pauropoda fits well with the Edafopoda hypothesis. The diplopodan postfemur and tibia might have derived by secondary annulation of a single podomere in a diplopodan ancestor. This interpretation is supported by the pattern of muscle origins at these podomeres. Independent of character polarisation, the number of podomeres support a split between Symphyla+Pauropoda and Chilopoda+Diplopoda.

Chilopoda and Diplopoda are mentioned to share a series of comb lamellae on the mandibles [63], lacking in Symphyla and maybe also in Pauropoda (for lamellar structures see [64]).

In summary, we assess the morphological evidence for Progoneata as weak. The evidence for Dignatha is much stronger but challenged by conflicting morphological signal supporting a split between Symphyla+Pauropoda and Chilopoda+Diplopoda. Morphological re-investigations with state-of-the-art methods are crucially needed to gain deeper insights in myriapod evolution and phylogeny. More comprehensive morphological data should then be supplemented by complete genome data from Symphyla and Pauropoda.

**Additional File 2: Supplementary Tables - Overview (szucsich-etal-MYRIAPOD PHYLOGENOMICS_Additional_File_2.xls)**

Table S1: Taxon sampling and accession numbers of raw and assembled transcriptome data of species included in this study.

Table S2: Collection information.

Table S3: Assembly statistics of published transcriptome data *de novo* assembled.

Table S4: Contamination and assembly statistics of de novo assembled transcriptome data newly sequenced for this study.

Table S5: Information and source of the reference species included in the ortholog set.

Table S6: Orthograph statistics.

Table S7: Group definitions to compile the data sets RELAXEDaa and RELAXEDnt (2^nd^ codon positions).

Table S8: Supermatrix diagnostics of final data sets compared with those analysed by Fernandez et al., 2018.

Table S9: Overview of statistical bootstrap and transfer bootstrap support of selected clades.

Table S10: Group definitions used for Four-cluster Likelihood Mapping (FcLM) analyses.

Table S11: FcLM results testing the position of Myriapoda within Euarthropoda (Mandibulata versus Paradoxopoda).

Table S12: Outgroup dependence: FcLM results testing myriapod relationships with Chelicerata and Onychophora as outgroup (data set STRICTaa_ChO).

Table S13. Outgroup dependence: FcLM results testing myriapod relationships with Pancrustacea as outgroup (data set STRICTaa_Pan).

Table S14: Amino acid and nucleotide frequencies of included species in data sets STRICTaa and STRICTnt.

**Additional File 3: Supplementary Figures - Overview (szucsich-etal-MYRIAPOD PHYLOGENOMICS_Additional_File_3.pdf)**

**Fig. S1. Heat maps calculated with *SymTest* applying the Bowker‘s test on data sets STRICT and RELAXED.**

The heatmaps show the results of pairwise Bowker’s test as implemented in *SymTest* 2.0.47 analysing the supermatrices STRICT and RELAXED. The percentage of pairwise p-values < 0.05 rejecting SRH conditions are given in parentheses. Data set STRICT: **a)** amino acids (p-values < 0.05: 88.43%), **b)** 1^st^ codon positions (p-values < 0.05: 99.3%), **c)** 2^nd^ codon positions (p-values < 0.05: 85.15%), **d)** 3^rd^ codon positions (p-values < 0.05: 100%). Data set RELAXED: **e)** amino acids (p-values < 0.05: 99.3%), **f)** 1^st^ codon positions (p-values < 0.05: 99.94%), **g)** 2^nd^ codon positions (p-values < 0.05: 96.9%), **h)** 3^rd^ codon positions (p-values < 0.05: 100%).

**Fig. S2. Heat maps visualising the information content (IC) of our final data sets STRICTaa and RELAXEDaa calculated with *Mare.***

The IC is color-coded in shades of blue, with darker shades representing higher IC and white squares indicate missing data, red squares (here not present) indicate meta-partitions with an IC = 0. **a)** data set STRICTaa. The 59 species are displayed in rows (x-axis) and the 215 meta-partitions (overall multiple sequence alignment length 95,797 amino acid sites) are shown in columns (y-axis). Overall information content: 0.303, matrix coverage in terms of meta-partitions: 100%. **b)** data set RELAXEDaa. The 59 species are displayed in rows (x-axis) and the 692 meta-partitions (overall multiple sequence alignment length 348,917 amino acid sites) are shown in columns (y-axis). Overall information content: 0.265, matrix coverage in terms of meta-partitions: 96.8%. Further diagnostics see Table S8.

**Fig. S3. Superalignment diagnostics of the data sets STRICTaa and RELAXEDaa.**

Heat maps indicating species‐pairwise amino acid site-coverage inferred with *AliStat* of the sequences of 59 species. Low shared site-coverage are in shades of red and high shared site-coverage in shades of green. **a)** data set STRICTaa: Completeness alignment score (Ca): 82.53%, Maximum C-score for individual sequences (Cr_max): 97.04%, Minimum C-score for individual sequences (Cr_min): 39.41%. **b)** data set RELAXEDaa: Ca: 72.13%, Cr_max: 95.89%, Cr_min: 32.33%. Further diagnostics in Table S8.

**Fig. S4. Heat map visualising the information content (IC) of matrix 1 of Fernandez et al. (2018) calculated with *Mare.***

The IC is color-coded in shades of blue, with darker shades representing higher IC and white squares indicate missing data. Red squares indicate gene partitions with an IC = 0. The 20 species are displayed in rows (x-axis) and the 229 gene partitions (overall multiple sequence alignment length 49,576 amino acid sites) are shown in columns (y-axis). Overall information content: 0.197, matrix coverage in terms of gene partitions: 78%. Further diagnostics, see Table S8.

**Fig. S5. Superalignment diagnostics of matrix 1 (Fernandez et al., 2018).**

The heat map indicates species‐pairwise amino-acid site coverage of matrix 1 (20 species, Fernandez et al., 2018) inferred with *AliStat*. Low shared site-coverage are in shades of red and high shared site- coverage are in shades of green. Completeness alignment score (Ca): 72.67%, Maximum C-score for individual sequences (Cr_max): 97.08%, Minimum C-score for individual sequences (Cr_min): 10.19%. Further diagnostics in Table S8.

**Fig. S6. Heat map calculated with *SymTest* applying the Bowker‘s test on matrix 1 (Fernandez et al., 2018).**

The heatmap shows the results of pairwise Bowker’s test as implemented in *SymTest* 2.0.47 analysing matrix 1 (amino acid level) of Fernandez et al. (2018). Percentage of pairwise p-values < 0.05 rejecting SRH conditions: 64.74%.

**Fig. S7. Best ML tree inferred from the data set STRICTaa with transfer bootstrap support.**

The ML tree is identical with the ML tree displayed in Fig. 2a with statistical transfer bootstrap support (TBE) inferred from all bootstrap trees with *Booster* v. 0.1.2. Values range from 0-1 (rounded to two decimal places). The tree was rooted with Onychophora.

**Fig. S8. Inferred ML tree from the data set STRICTaa with the CAT-like mixture model + PSMF.**

Inferred ML tree from the data set STRICTaa using the unpartitioned approach applying the CAT-like mixture model + PSMF with statistical non-parametric bootstrap support inferred from 100 replicates. The tree was rooted with Onychophora.

**Fig. S9. Inferred ML tree from the data set STRICTaa with theCAT-like mixture model + PSMF with transfer bootstrap support.**

The ML tree is identical to the ML tree displayed in Fig. S8 with statistical transfer bootstrap support (TBE) inferred from all bootstrap trees with *Booster* v. 0.1.2. Values range from 0-1 (rounded to two decimal places). The tree was rooted with Onychophora.

**Fig. S10. Best ML tree inferred from the data set RELAXEDaa.**

Statistical non-parametric bootstrap support was inferred from 100 replicates. The tree was rooted with Onychophora.

**Fig. S11. Best ML tree inferred from the data set RELAXEDaa with transfer bootstrap support.**

The ML tree is identical to the ML tree displayed in Fig. S10 with statistical transfer bootstrap support (TBE) inferred from all bootstrap trees with *Booster* v. 0.1.2. Values range from 0-1 (rounded to two decimal places). The tree was rooted with Onychophora.

**Fig. S12. Inferred ML tree from the data set RELAXEDaa with the CAT-like mixture model + PSMF.**

Inferred ML tree from the data set RELAXEDaa using the unpartitioned approach applying the CAT-like mixture model + PSMF with statistical non-parametric bootstrap support inferred from 100 replicates. The tree was rooted with Onychophora.

**Fig. S13. Inferred ML tree from the data set RELAXEDaa with the CAT-like mixture model + PSMF with transfer bootstrap support.**

The ML tree is identical to the ML tree displayed in Fig. S12 with statistical transfer bootstrap support (TBE) inferred from all bootstrap trees with *Booster* v. 0.1.2. Values range from 0-1 (rounded to two decimal places). The tree was rooted with Onychophora.

**Fig. S14. Best ML tree inferred from the data set STRICTnt.**

Data set STRICTnt only includes 2^nd^ codon positions. Statistical non-parametric bootstrap support was inferred from 100 replicates. The tree was rooted with Onychophora.

**Fig. S15. Best ML tree inferred from the data set STRICTnt with transfer bootstrap support.**

The ML tree is identical to the ML tree displayed in Fig. S14 with statistical transfer bootstrap support (TBE) inferred from all bootstrap trees with *Booster* v. 0.1.2. Values range from 0-1 (rounded to two decimal places). The tree was rooted with Onychophora.

**Fig. S16. Best ML tree inferred from the data set RELAXEDnt with non-parametric statistical bootstrap support.**

Data set RELAXEDnt only includes 2^nd^ codon positions. Statistical non-parametric bootstrap support was inferred from 100 replicates. The tree was rooted with Onychophora.

**Fig. S17. Best ML tree inferred from the data set RELAXEDnt with transfer bootstrap support.**

The ML tree is identical to the ML tree displayed in Fig. S16 with statistical transfer bootstrap support (TBE) inferred from all bootstrap trees with *Booster* v. 0.1.2. Values range from 0-1 (rounded to two decimal places). The tree was rooted with Onychophora.

**Fig. S18. Best ML tree inferred from the data set STRICTaa_ChO**

Data set STRICTaa_ChO includes only Chelicerata and Onychophora as outgroup (excluding Pancrustacea). Statistical non-parametric bootstrap support was inferred from 100 replicates. The tree was rooted with Onychophora.

**Fig. S19. Best ML tree inferred from the data set STRICTaa_Pan.**

Data set STRICTaa_Pan includes only Pancrustacea as outgroup (excluding Chelicerata and Onychophora). Statistical non-parametric bootstrap support was inferred from 100 replicates. The tree was rooted with Pancrustacea.

**Supplementary Archives in DRYAD (doi:10.5061/dryad.cvdncjt2r)**

Supplementary Archive 1: *De novo* assembled transcriptomes of published raw sequence reads (*Peripatopsis capensis*, *Eupolybothrus cavernicolus*, *Cryptops hortensis*)

Supplementary Archive 2: Ortholog Set: official gene sets of four reference species (see Table S5) on protein and nucleotide level and table of ortholog sequence groups [ORTHOGRAPH format]

Supplementary Archive 3: Optimised data sets [FASTA format] with the best partition schemes and selected models [NEXUS format]: STRICTaa and STRICTnt (2^nd^ codon positions), RELAXEDaa and RELAXEDnt (2^nd^ codon positions)

Supplementary Archive 4: Best ML trees with statistical support from non-parametric bootstraps and transfer bootstrap support for data sets STRICTaa inferred with the partitioned approach, STRICTaa inferred with the CAT-like mixture model + PMSF, STRICTnt (2^nd^ codon positions) inferred with the partitioned approach, RELAXEDaa inferred with the partitioned approach, RELAXEDaa inferred with the CAT-like mixture model + PMSF and RELAXEDnt (2^nd^ codon positions) [NEWICK format]

Supplementary Archive 5: FcLM data sets designed to test the position of Myriapoda within euarthropods (Mandibulata vs. Paradoxopoda) (Table S11): data sets [FASTA format] and respective partition schemes with selected models (original + permutation I, II, III)

Supplementary Archive 6: FcLM data sets designed to test relationships among myriapod subgroups (Table 2): data sets [FASTA format] and respective partition schemes with selected models (original + permutation I, II, III)

Supplementary Archive 7: testing outgroup dependence of relationships among myriapod subgroups (outgroup restricted to Chelicerata+Onychophora)

Supplementary Archive 7a: data set [FASTA format], partition scheme with selected model [NEXUS format] & best ML tree with Bootstrap support [NEWICK format] (outgroup restricted to Chelicerata+Onychophora)

Supplementary Archive 7b: FcLM data subsets [FASTA format] in each subset one myriapod subgroup is excluded (Table S12) - partition schemes with selected models (original + permutation I, II, III) (outgroup restricted to Chelicerata+Onychophora)

Supplementary Archive 8: testing outgroup dependence of relationships among myriapod subgroups (outgroup restricted to Pancrustacea)

Supplementary Archive 8a: data set [FASTA format], partition scheme with selected model [NEXUS format] & best ML tree with Bootstrap support [NEWICK format] (outgroup restricted to Pancrustacea)

Supplementary Archive 8b: FcLM data subsets [FASTA format] in each subset one myriapod subgroup is excluded (Table S13) - partition schemes with selected models (original + permutation I, II, III) (outgroup restricted to Pancrustacea)

**Supplementary References**

1. Misof B, Liu S, Meusemann K, Peters RS, Donath A, Mayer C et al. Phylogenomics resolves the timing and pattern of insect evolution. Science. 2014;346:763-7; <http://dx.doi.org/10.1126/science.1257570>
2. Peters RS, Krogmann L, Mayer C, Donath A, Gunkel S, Meusemann K et al. Evolutionary history of the Hymenoptera. Curr Biol. 2017;27:1013-8; <https://doi.org/10.1016/j.cub.2017.01.027>
3. Stoev P, Komerički MA, Akkari N, Liu MS, Zhou MX, Weigand AM et al. *Eupolybothrus cavernicolus* Komerički & Stoev sp. n. (Chilopoda: Lithobiomorpha: Lithobiidae): the first eukaryotic species description combining transcriptomic, DNA barcoding and micro-CT imaging data. Biodiversity Data J. 2013; <https://dx.doi.org/10.3897%2FBDJ.1.e1013>
4. Fernández R, Laumer CE, Vahtera V, Libro S, Kaluziak S, Sharma PP et al. Evaluating topological conflict in centipede phylogeny using transcriptomic data sets. Mol Biol Evol. 2014;31:1500-13. <https://doi.org/10.1093/molbev/msu108>
5. Grabherr MG, Haas BJ, Yassour M, Levin JZ, Thompson DA, Amit I et al. Full-length transcriptome assembly from RNA-Seq data without a reference genome. Nature Biotechnology. 2011;29:644; <https://doi.org/10.1038/nbt.1883>
6. Haas BJ, Papanicolaou A, Yassour M, Grabherr M, Blood PD, Bowden J et al. De novo transcript sequence reconstruction from RNA-seq using the Trinity platform for reference generation and analysis. Nature Protocols. 2013;8:1494; <https://doi.org/10.1038/nprot.2013.084>
7. Xie Y, Wu G, Tang J, Luo R, Patterson J, Liu S et al. SOAPdenovo-Trans: de novo transcriptome assembly with short RNA-Seq reads. Bioinformatics. 2014;30:1660-6; https://doi.org/10.1093/bioinformatics/btu077
8. Mayer C, Sann M, Donath A, Meixner M, Podsiadlowski L, Peters RS et al. BaitFisher: a software package for multispecies target DNA enrichment probe design. Mol Biol Evol. 2016;33:1875-86; <https://doi.org/10.1093/molbev/msw056>
9. Camacho C, Coulouris G, Avagyan V, Ma N, Papadopoulos J, Bealer K et al. BLAST+: architecture and applications. BMC Bioinformatics. 2009;10:421; <https://doi.org/10.1186/1471-2105-10-421>
10. Kriventseva EV, Tegenfeldt F, Petty TJ, Waterhouse RM, Simao FA, Pozdnyakov IA et al. OrthoDB v8: update of the hierarchical catalog of orthologs and the underlying free software. Nucleic Acids Res. 2015;43:D250-D6; <https://doi.org/10.1093/nar/gku1220>
11. Petersen M, Meusemann K, Donath A, Dowling D, Liu S, Peters RS et al. Orthograph: a versatile tool for mapping coding nucleotide sequences to clusters of orthologous genes. BMC Bioinformatics. 2017;18:1-10; <https://doi.org/10.1186/s12859-017-1529-8>
12. Katoh K, Standley DM. MAFFT multiple sequence alignment software version 7: improvements in performance and usability. Mol Biol Evol. 2013;30:772-80; <https://doi.org/10.1093/molbev/mst010>
13. Suyama M, Torrents D, Bork P. PAL2NAL: robust conversion of protein sequence alignments into the corresponding codon alignments. Nucleic Acids Res. 2006;34:W609-W12; <https://doi.org/10.1093/nar/gkl315>
14. Misof B, Misof K. A Monte Carlo approach successfully identifies randomness in multiple sequence alignments: a more objective means of data exclusion. Syst Biol. 2009;58:21-34; <https://doi.org/10.1093/sysbio/syp006>
15. Kück P, Meusemann K, Dambach J, Thormann B, von Reumont BM, Wägele JW et al. Parametric and non-parametric masking of randomness in sequence alignments can be improved and leads to better resolved trees. Front Zool. 2010;7:10; <https://doi.org/10.1186/1742-9994-7-10>
16. Kück P, Meusemann K. FASconCAT: convenient handling of data matrices. Mol Phylogenet Evol. 2010;56:1115-8; <https://doi.org/10.1016/j.ympev.2010.04.024>
17. Misof B, Meyer B, von Reumont BM, Kück P, Misof K, Meusemann K. Selecting informative subsets of sparse supermatrices increases the chance to find correct trees. BMC Bioinformatics. 2013;14:348; <https://doi.org/10.1186/1471-2105-14-348>
18. Dell’Ampio E, Meusemann K, Szucsich NU, Peters RS, Meyer B, Borner J et al. Decisive data sets in phylogenomics: lessons from studies on the phylogenetic relationships of primarily wingless insects. Mol Biol Evol. 2014;31:239-49; <https://doi.org/10.1093/molbev/mst196>
19. Jermiin L, Ott M. SymTest version 2.0.47 2017. (<https://github.com/ottmi/symtest>).
20. Ho SY, Jermiin LS. Tracing the decay of the historical signal in biological sequence data. Syst Biol. 2004;53:623-37; <https://doi.org/10.1080/10635150490503035>
21. Jermiin LS, Ho SY, Ababneh F, Robinson J, Larkum AW. The biasing effect of compositional heterogeneity on phylogenetic estimates may be underestimated. Syst Biol. 2004;53:638-43; <https://doi.org/10.1080/10635150490468648>
22. Bowker AH. A test for symmetry in contingency tables. J Am Stat Assoc. 1948;43:572-4; <https://doi.org/10.1080/01621459.1948.10483284>
23. Simon S, Blanke A, Meusemann K. Reanalyzing the Palaeoptera problem–the origin of insect flight remains obscure. Arthropod Struc Dev. 2018;47:328-38; <https://doi.org/10.1016/j.asd.2018.05.002>
24. Lanfear R, Frandsen PB, Wright AM, Senfeld T, Calcott B. PartitionFinder 2: new methods for selecting partitioned models of evolution for molecular and morphological phylogenetic analyses. Mol Biol Evol. 2017;34:772-3; <https://doi.org/10.1093/molbev/msw260>
25. Stamatakis A. RAxML version 8: a tool for phylogenetic analysis and post-analysis of large phylogenies. Bioinformatics. 2014;30:1312-3; <https://doi.org/10.1093/bioinformatics/btu033>
26. Le SQ, Gascuel O: An improved general amino acid replacement matrix. Mol Biol Evol 2008, 25:1307-1320. <https://doi.org/10.1093/molbev/msn067>
27. Whelan S, Goldman N. A general empirical model of protein evolution derived from multiple protein families using a maximum-likelihood approach. Mol Biol Evol. 2001;18:691-9; <https://doi.org/10.1093/oxfordjournals.molbev.a003851>
28. Kosiol C, Goldman N. Different versions of the Dayhoff rate matrix. Mol Biol Evol. 2005;22:193-9; <https://doi.org/10.1093/molbev/msi005>
29. Jones DT, Taylor WR, Thornton JM. The rapid generation of mutation data matrices from protein sequences. Bioinformatics. 1992;8:275-82; <https://doi.org/10.1093/bioinformatics/8.3.275>
30. Henikoff S, Henikoff JG. Amino acid substitution matrices from protein blocks. Proc Natl Acad Sci USA. 1992;89:10915-9; <https://doi.org/10.1073/pnas.89.22.10915>
31. Le SQ, Dang CC, Gascuel O. Modeling protein evolution with several amino acid replacement matrices depending on site rates. Mol Biol Evol. 2012;29:2921-36; <https://doi.org/10.1093/molbev/mss112>
32. Hurvich CM, Tsai C-L. Regression and time series model selection in small samples. Biometrika. 1989;76:297-307; <https://doi.org/10.1093/biomet/76.2.297>
33. Wong TKF, Kalyaanamoorthy S, Meusemann K, Yeates DK, Misof B, Jermiin LS: A minimum reporting standard for multiple sequence alignments. NAR Genomics and Bioinformatics 2020, 2:lqaa024. <https://doi.org/10.1093/nargab/lqaa024>
34. Fernández R, Edgecombe GD, Giribet G. Phylogenomics illuminates the backbone of the Myriapoda Tree of Life and reconciles morphological and molecular phylogenies. Sci Rep. 2018;8; <https://doi.org/10.1038/s41598-017-18562-w>
35. Nguyen L-T, Schmidt HA, Von Haeseler A, Minh BQ. IQ-TREE: a fast and effective stochastic algorithm for estimating maximum-likelihood phylogenies. Mol Biol Evol. 2015;32:268-74; <https://doi.org/10.1093/molbev/msu300>
36. Chernomor O, von Haeseler A, Minh BQ. Terrace aware data structure for phylogenomic inference from supermatrices. Syst Biol. 2016;65:997-1008; <https://doi.org/10.1093/sysbio/syw037>
37. Pattengale ND, Alipour M, Bininda-Emonds OR, Moret BM, Stamatakis A, editors. How many bootstrap replicates are necessary? Annual International Conference on Research in Computational Molecular Biology; 2009: Springer.
38. Lemoine F, Entfellner J-BD, Wilkinson E, Correia D, Felipe MD, De Oliveira T et al. Renewing Felsenstein’s phylogenetic bootstrap in the era of big data. Nature. 2018;556:452-6; <https://doi.org/10.1038/s41586-018-0043-0>
39. Aberer AJ, Krompass D, Stamatakis A. Pruning rogue taxa improves phylogenetic accuracy: an efficient algorithm and webservice. Syst Biol. 2013;62:162-6; <https://doi.org/10.1093/sysbio/sys078>
40. Lartillot N, Philippe H. A Bayesian mixture model for across-site heterogeneities in the amino-acid replacement process. Mol Biol Evol. 2004;21:1095-109; <https://doi.org/10.1093/molbev/msh112>
41. Nascimento FF, dos Reis M, Yang Z. A biologist’s guide to Bayesian phylogenetic analysis. Nat Ecol Evol. 2017;1:1446-54; <https://doi.org/10.1038/s41559-017-0280-x>
42. Schwentner M, Combosch DJ, Nelson JP, Giribet G. A phylogenomic solution to the origin of insects by resolving crustacean-hexapod relationships. Curr Biol. 2017;27:1818-24. e5; <https://doi.org/10.1016/j.cub.2017.05.040>
43. Whelan NV, Halanych KM. Who let the CAT out of the bag? Accurately dealing with substitutional heterogeneity in phylogenomic analyses. Syst Biol. 2017;66:232-55; <https://doi.org/10.1093/sysbio/syw084>
44. Blanquart S, Lartillot N: A site- and time-heterogeneous model of amino acid replacement. Mol Biol Evol 2008, 25:842-858. <https://doi.org/10.1093/molbev/msn018>
45. Quang LS, Gascuel O, Lartillot N. Empirical profile mixture models for phylogenetic reconstruction. Bioinformatics. 2008;24:2317-23; <https://doi.org/10.1093/bioinformatics/btn445>
46. Wang H-C, Minh BQ, Susko E, Roger AJ. Modeling site heterogeneity with posterior mean site frequency profiles accelerates accurate phylogenomic estimation. Syst Biol. 2018;67:216-35; <https://doi.org/10.1093/sysbio/syx068>
47. Gouy M, Guindon S, Gascuel O. SeaView version 4: a multiplatform graphical user interface for sequence alignment and phylogenetic tree building. Mol Biol Evol. 2010;27:221-4; <https://doi.org/10.1093/molbev/msp259>
48. Shimodaira H. An approximately unbiased test of phylogenetic tree selection. Syst Biol. 2002;51:492-508; <https://doi.org/10.1080/10635150290069913>
49. Maddison W, Maddison D. Mesquite: a modular system for evolutionary analysis. Version 3.10. 2016. 2016.
50. Regier JC, Shultz JW, Zwick A, Hussey A, Ball B, Wetzer R et al. Arthropod relationships revealed by phylogenomic analysis of nuclear protein-coding sequences. Nature. 2010;463:1079-83; <https://doi.org/10.1038/nature08742>
51. Zwick A, Regier JC, Zwickl DJ. Resolving discrepancy between nucleotides and amino acids in deep-level arthropod phylogenomics: differentiating serine codons in 21-amino-acid models. PLoS One. 2012;7; <https://doi.org/10.1371/journal.pone.0047450>
52. Edgecombe GD. 1 Phylogenetic relationships of Myriapoda. Treatise on Zoology-Anatomy, Taxonomy, Biology The Myriapoda. 1: Brill; 2011. p. 1-20.
53. Tiegs OW. The development and affinities of the Pauropoda, based on a study of *Pauropus silvaticus*. Q J Microsc Sci. 1947;88:275-336; https://jcs.biologists.org/content/s3-88/3/275
54. Strimmer K, Von Haeseler A. Likelihood-mapping: a simple method to visualize phylogenetic content of a sequence alignment. Proc Natl Acad Sci USA. 1997;94:6815-9; <https://doi.org/10.1073/pnas.94.13.6815>
55. Dunlop,JA, Lamsdell JC. Segmentation and tagmosis in Chelicerata. Arthropod Struct Dev 2017;46:395-418.
56. Abzhanov A, Holtzman S, Kaufman TC. The *Drosophila* proboscis is specified by two Hox genes, proboscipedia and Sex combs reduced, via repression of leg and antennal appendage genes. Development 2001;128:2803-2814.
57. Dohle W. Myriapod-insect relationships as opposed to an insect-crustacean sister group relationship. In: Arthropod Relationships. Fortey RA, Thomas, RH (eds). London, Chapman & Hall: 1997;305-315.
58. Scheller U. A reclassification of the Pauropoda (Myriapoda). Int J Myriap 2008;1:1-38. <https://doi.org/10.1163/187525408X316730>
59. Szucsich NU, Scheller U. Chapter 20 Symphyla. Treatise of Zoology – Anatomy; Taxonomy, Biology – The Myriapoda 1. A. Minelli. Leiden, NL, Brill 2011:445-466.
60. Pennerstorfer M. The anatomy of the locomotory apparatus of *Scutigerella* sp. (Symphyla, „Myriapoda“): a re-investigation and comparison to other myriapods. Department of Evolutionary Biology. Vienna, University of Vienna: 2007 (unpubl.):pp113.
61. Manton SM. The Arthropoda, habits, functional morphology and evolution. Oxford, Claredon Press 1977:pp527.
62. Dunger W. Antennata. In: Lehrbuch der Speziellen Zoologie. Gruner HE (ed). Jena, Gustav Fischer Verlag. 1993:1(4, 4):1031-1160.
63. Edgecombe GD, Giribet G. Myriapod phylogeny and the relationships of Chilopoda. Biodiversidad, In: Taxonomía y Biogeografía de Artropodos de México: Hacia una Síntesis de su Conocimiento. Llorente Bousquets JE, Morrone JJ (eds), Universidad Nacional Autonoma de México. 2002;III:143-168.
64. Hüther W. Erstnachweis der Pauropoda Hexamerocerata für Südamerika, mit Beschreibung einer neuen Art. Revue d’Écologie et de Biologie du Sol 1968;5:561-567.
