# Supplementary material for: Four myriapod relatives – but who are sisters? No end to debates on relationships among the four major myriapod subgroups
Source: BMC Evol Biol. 2020 Nov 4;20:144. doi: 10.1186/s12862-020-01699-0 (PMC7640414; doi:10.1186/s12862-020-01699-0)
Supplement: Supplementary file 3 — Additional file 3: Fig. S1. Heat maps calculated with SymTest applying the Bowker‘s test on data sets STRICT and RELAXED. The heatmaps show the results of pairwise Bowker’s test as implemented in SymTest 2.0.47 analysing the supermatrices STRICT and RELAXED. The percentage of pairwise p-values < 0.05 rejecting SRH conditions are given in parentheses. Data set STRICT: a) amino acids (p-values < 0.05: 88.43%), b) 1st codon positions (p-values < 0.05: 99.3%), c) 2nd codon positions (p-values < 0.05: 85.15%), d) 3rd codon positions (p-values < 0.05: 100%). Data set RELAXED: e) amino acids (p-values < 0.05: 99.3%), f) 1st codon positions (p-values < 0.05: 99.94%), g) 2nd codon positions (p-values < 0.05: 96.9%), h) 3rd codon positions (p-values < 0.05: 100%). Fig. S2. Heat maps visualising the information content (IC) of our final data sets STRICTaa and RELAXEDaa calculated with Mare. The IC is color-coded in shades of blue, with darker shades representing higher IC and white squares indicate missing data, red squares (here not present) indicate meta-partitions with an IC = 0. a) data set STRICTaa. The 59 species are displayed in rows (x-axis) and the 215 meta-partitions (overall multiple sequence alignment length 95,797 amino acid sites) are shown in columns (y-axis). Overall information content: 0.303, matrix coverage in terms of meta-partitions: 100%. b) data set RELAXEDaa. The 59 species are displayed in rows (x-axis) and the 692 meta-partitions (overall multiple sequence alignment length 348,917 amino acid sites) are shown in columns (y-axis). Overall information content: 0.265, matrix coverage in terms of meta-partitions: 96.8%. Further diagnostics see Table S8. Fig. S3. Superalignment diagnostics of the data sets STRICTaa and RELAXEDaa. Heat maps indicating species-pairwise amino acid site-coverage inferred with AliStat of the sequences of 59 species. Low shared site-coverage are in shades of red and high shared site-coverage in shades of green. a) data set STRICT [file 12862_2020_1699_MOESM3_ESM.pdf]

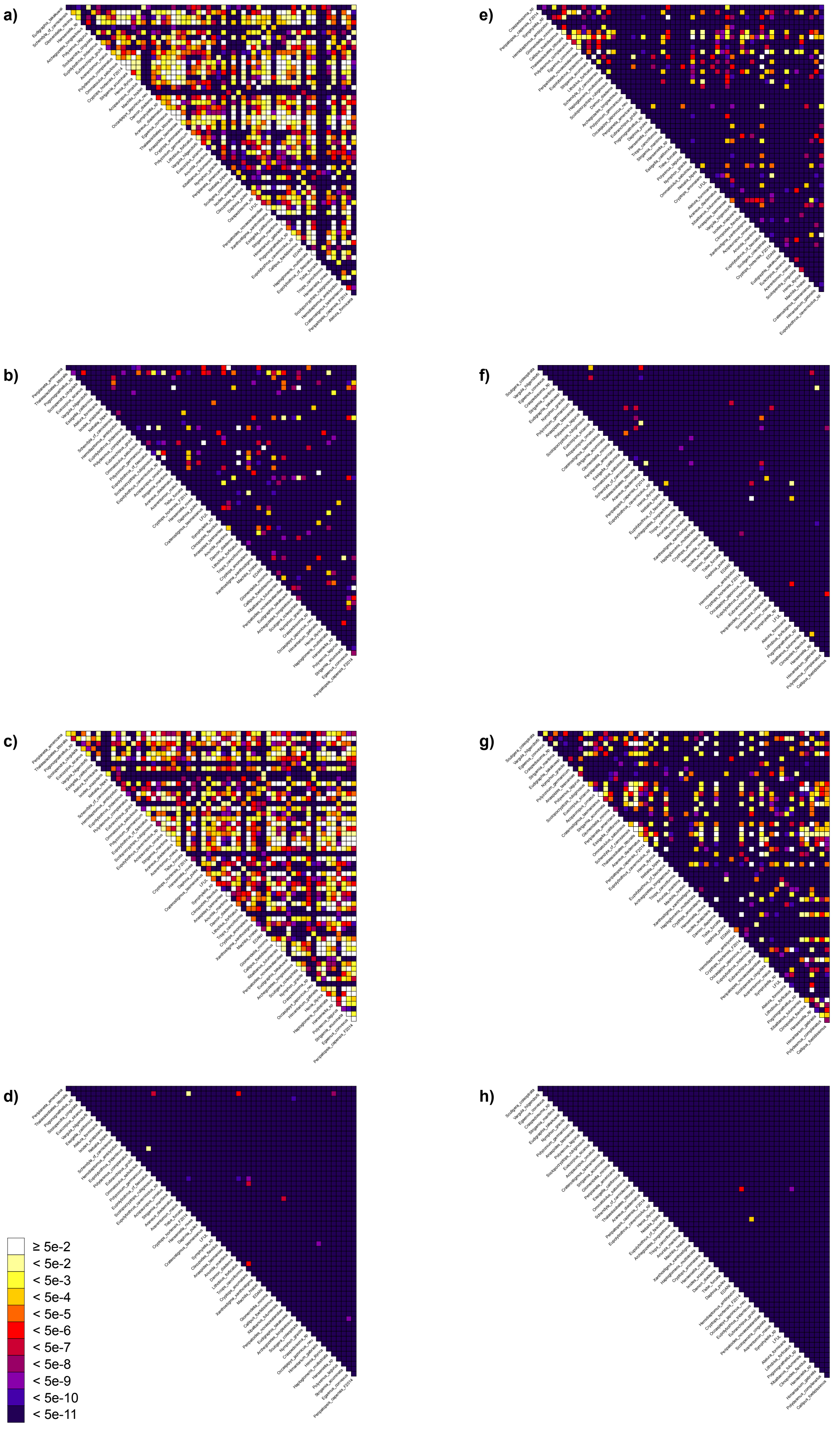

Fig. S1. Heat maps calculated with *SymTest* applying the Bowker's test of data sets STRICT and RELAXED. The heatmaps show the results of pairwise Bowker's test as implemented in *SymTest* 2.0.47 analysing the supermatrices STRICT and RELAXED. The percentage of pairwise p-values < 0.05 rejecting SRH conditions are given in parantheses. Data set STRICT: **a)** amino acids (p-values < 0.05: 88.43%), **b)** 1st codon positions (p-values < 0.05: 99.3%), **c)** 2nd codon codon positions (p-values < 0.05: 85.15%), **d)** 3rd codon positions (p-values < 0.05: 100%). Data set RELAXED: **e)** amino acids (p-values < 0.05: 99.3%), **f)** 1st codon positions (p-values < 0.05: 99.94%), **g)** 2nd codon positions (p-values < 0.05: 96.9%), **h)** 3rd codon positions (p-values < 0.05: 100%).

a) Data set STRICTaa

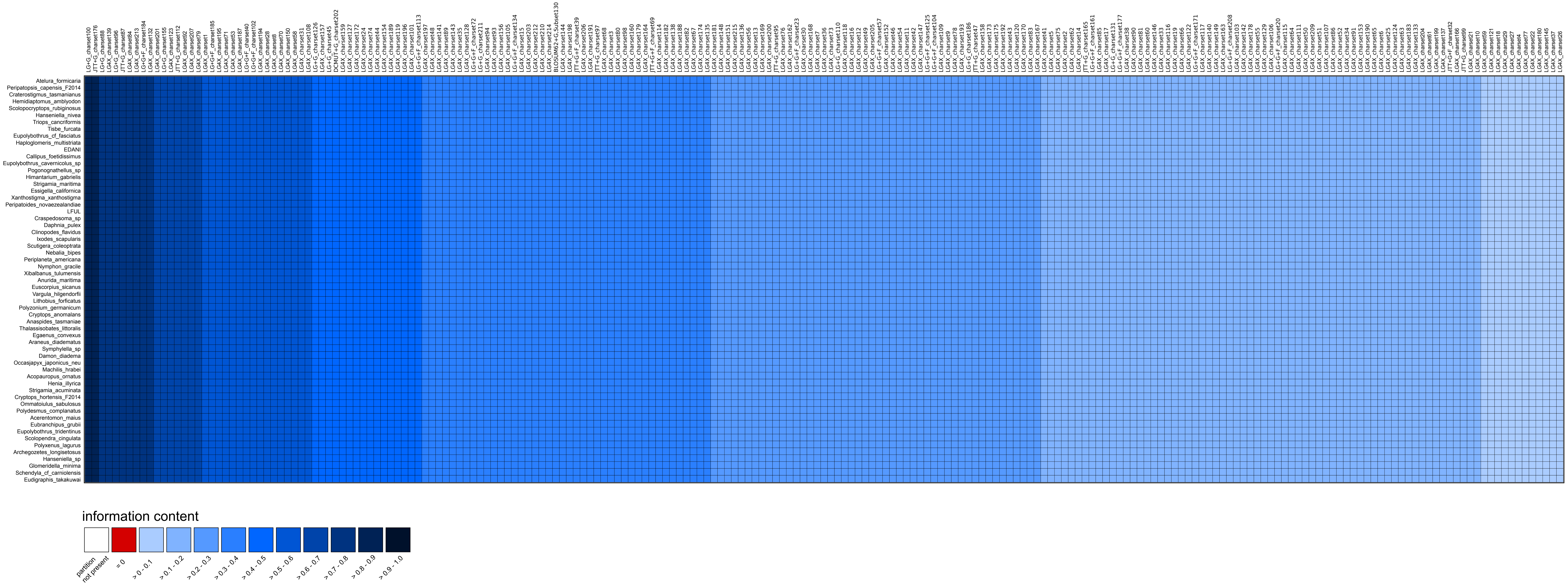

b) Data set RELAXEDaa

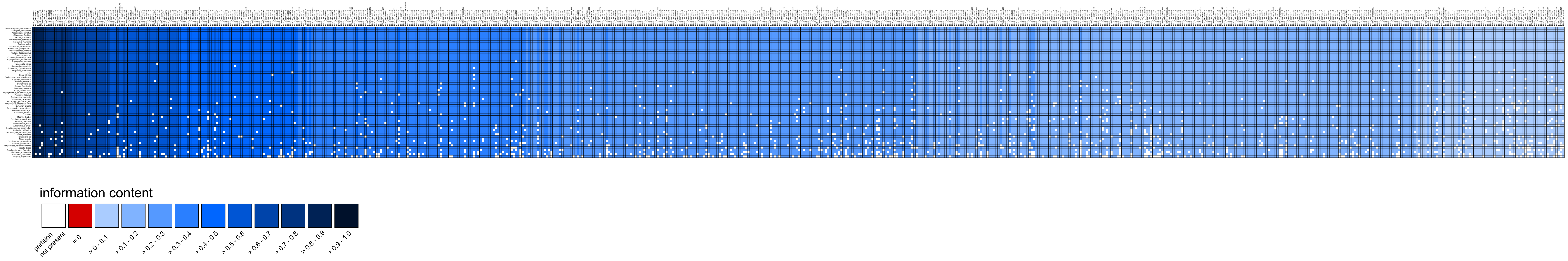

Fig. S2. Heat maps visualising the information content (IC) of our final data sets STRICTaa and RELAXEDaa calculated with *Mare*.
The IC is color-coded in shades of blue, with darker shades representing higher IC and white squares indicate missing data, red squares (here not present) indicate meta-partitions with an IC = 0. **a)** data set STRICTaa. The 59 species are displayed in rows (x-axis) and the 215 meta-partitions (overall multiple sequence alignment length 95,797 amino acid sites) are shown in columns (y-axis). Overall information content: 0.303, matrix coverage in terms of meta-partitions: 100%. **b)** data set RELAXEDaa. The 59 species are displayed in rows (x-axis) and the 692 meta-partitions (overall multiple sequence alignment length 348,917 amino acid sites) are shown in columns (y-axis). Overall information content: 0.265, matrix coverage in terms of meta-partitions: 96.8%. Further diagnostics see Table S8.

a)

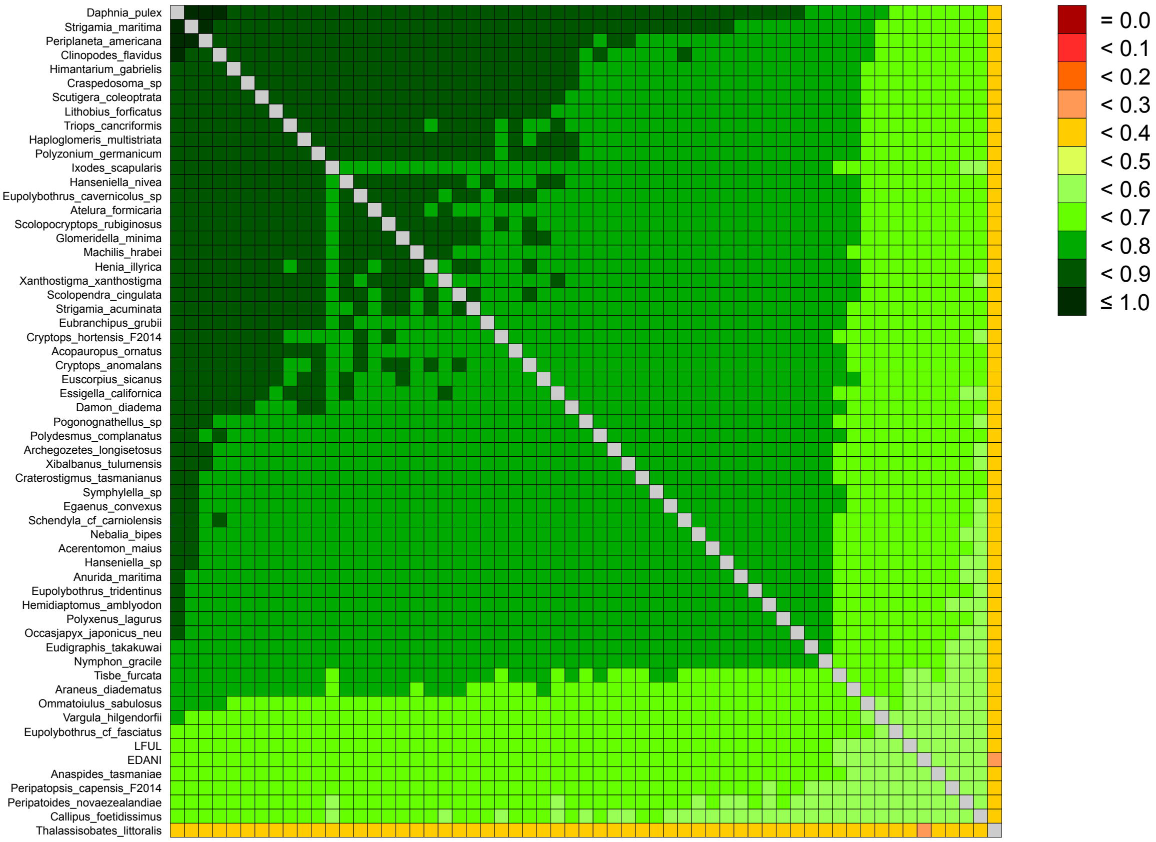

b)

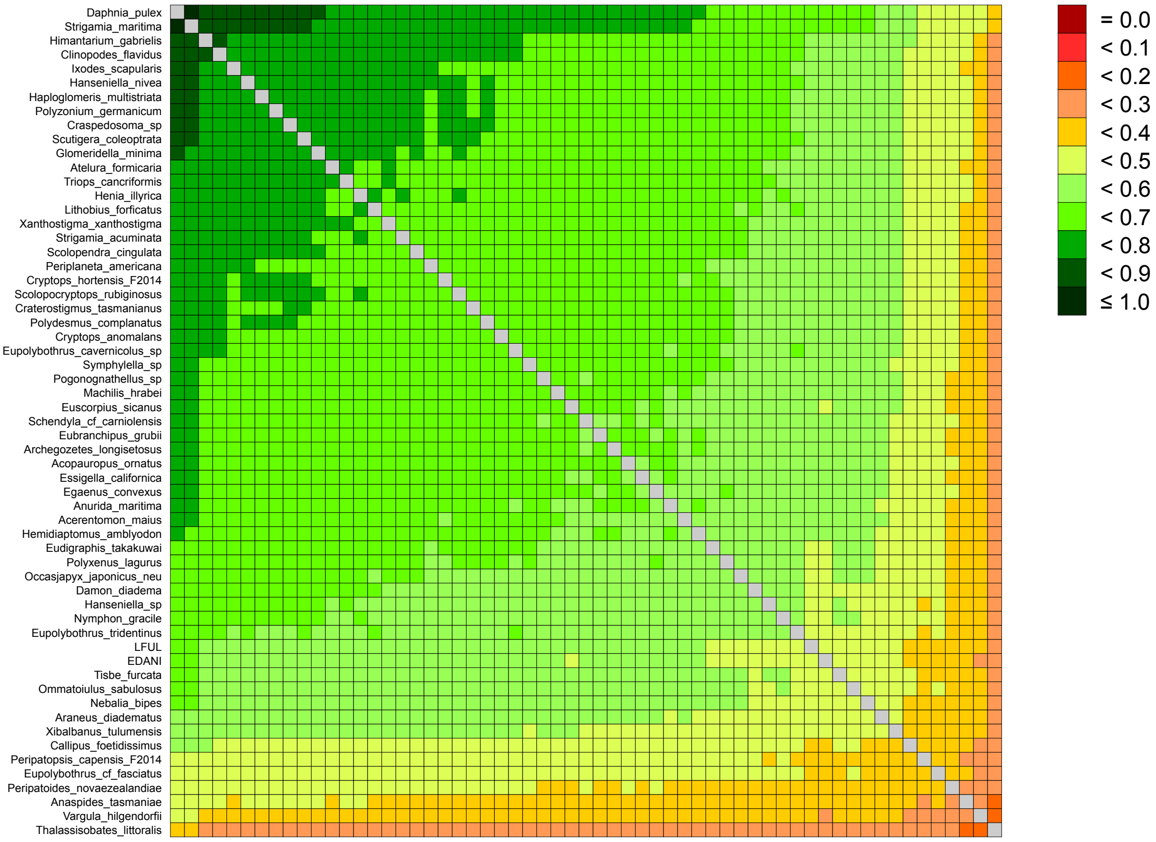

Fig. S3. Superalignment diagnostics of the data sets STRICTaa and RELAXEDaa. Heat maps indicating species-pairwise amino acid site-coverage inferred with *AliStat* of the sequences of 59 species. Low shared site-coverage are in shades of red and high shared site-coverage are in shades of green. **a)** data set STRICTaa: Completeness alignment score (Ca): 82.53%, Maximum C-score for individual sequences (Cr_max): 97.04%, Minimum C-score for individual sequences (Cr_min): 39.41%. **b)** data set RELAXEDaa: Ca: 72.13%, Cr_max: 95.89%, Cr_min: 32.33%. Further diagnostics in Table S8.

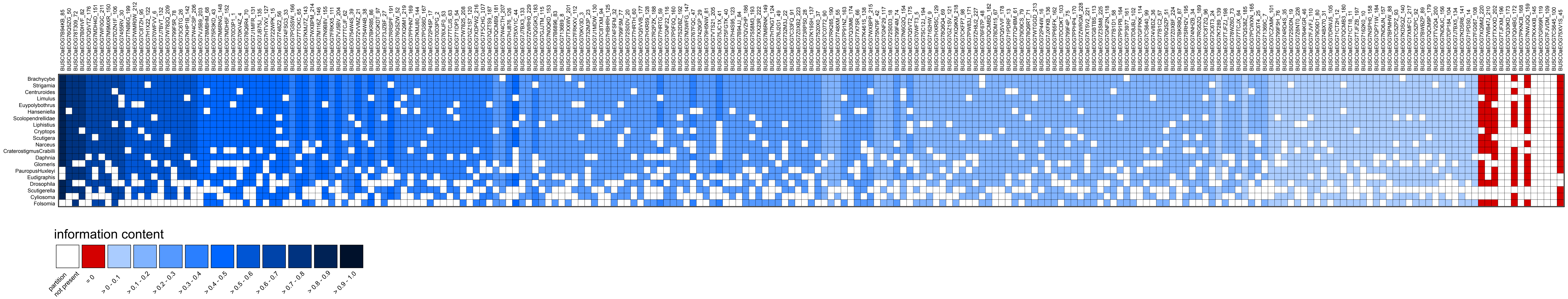

Fig. S4. Heat map visualising the information content (IC) of matrix 1 (amino acid level) of Fernandez et al. (2018) calculated with *Mare*.

The IC is color-coded in shades of blue, with darker shades representing higher IC and white squares indicate missing data. Red squares indicate gene partitions with an IC = 0. The 20 species are displayed in rows (x-axis) and the 229 gene partitions (overall multiple sequence alignment length 49,576 amino acid sites) are shown in columns (y-axis). Overall information content: 0.197, matrix coverage in terms of gene partitions: 78%. Further diagnostics, see Table S8.

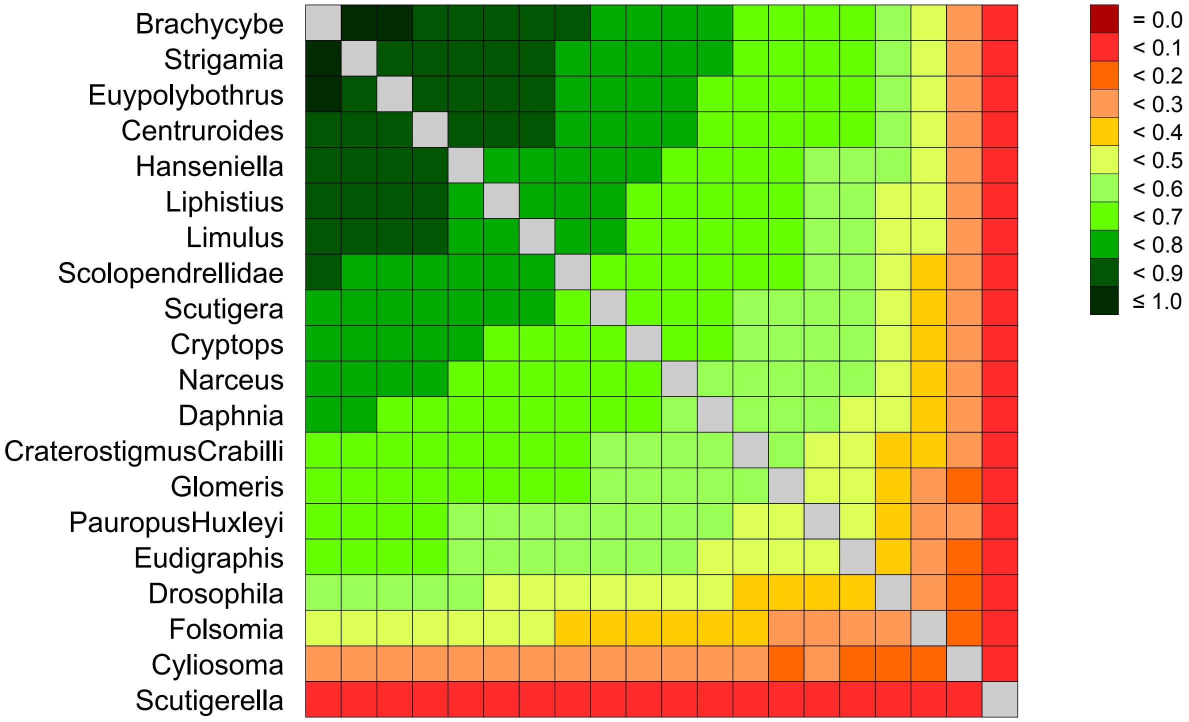

Fig. S5. Superalignment diagnostics of matrix 1 (Fernandez et al., 2018).
The heat map indicates species-pairwise amino-acid site coverage of matrix 1 (20 species, Fernandez et al., 2018) inferred with *AliStat*. Low shared site-coverage are in shades of red, high shared site-coverage are in shades of green. Completeness alignment score (Ca): 72.67%, Maximum C-score for individual sequences (Cr_max): 97.08%, Minimum C-score for individual sequences (Cr_min): 10.19%. Further diagnostics of matrix 1, 2 and 3 analysed by Fernandez et al. (2018) in Table S8.

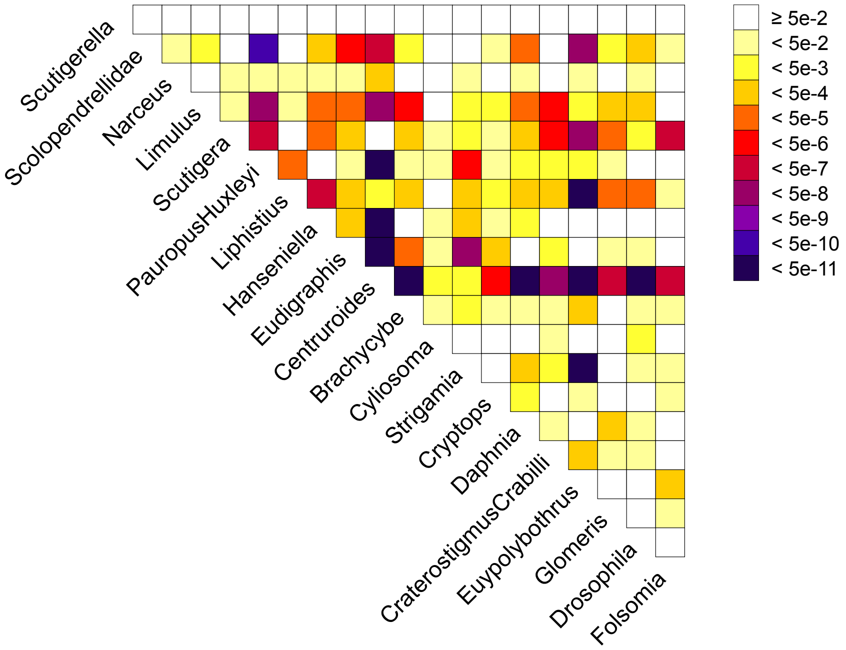

Fig. S6. Heat map calculated with *SymTest* applying the Bowker's test of matrix 1 (Fernandez et al., 2018).
The heatmap shows the results of pairwise Bowker's test as implemented in *SymTest 2.0.47* analysing matrix 1 (amino acid level) of Fernandez et al. (2018).

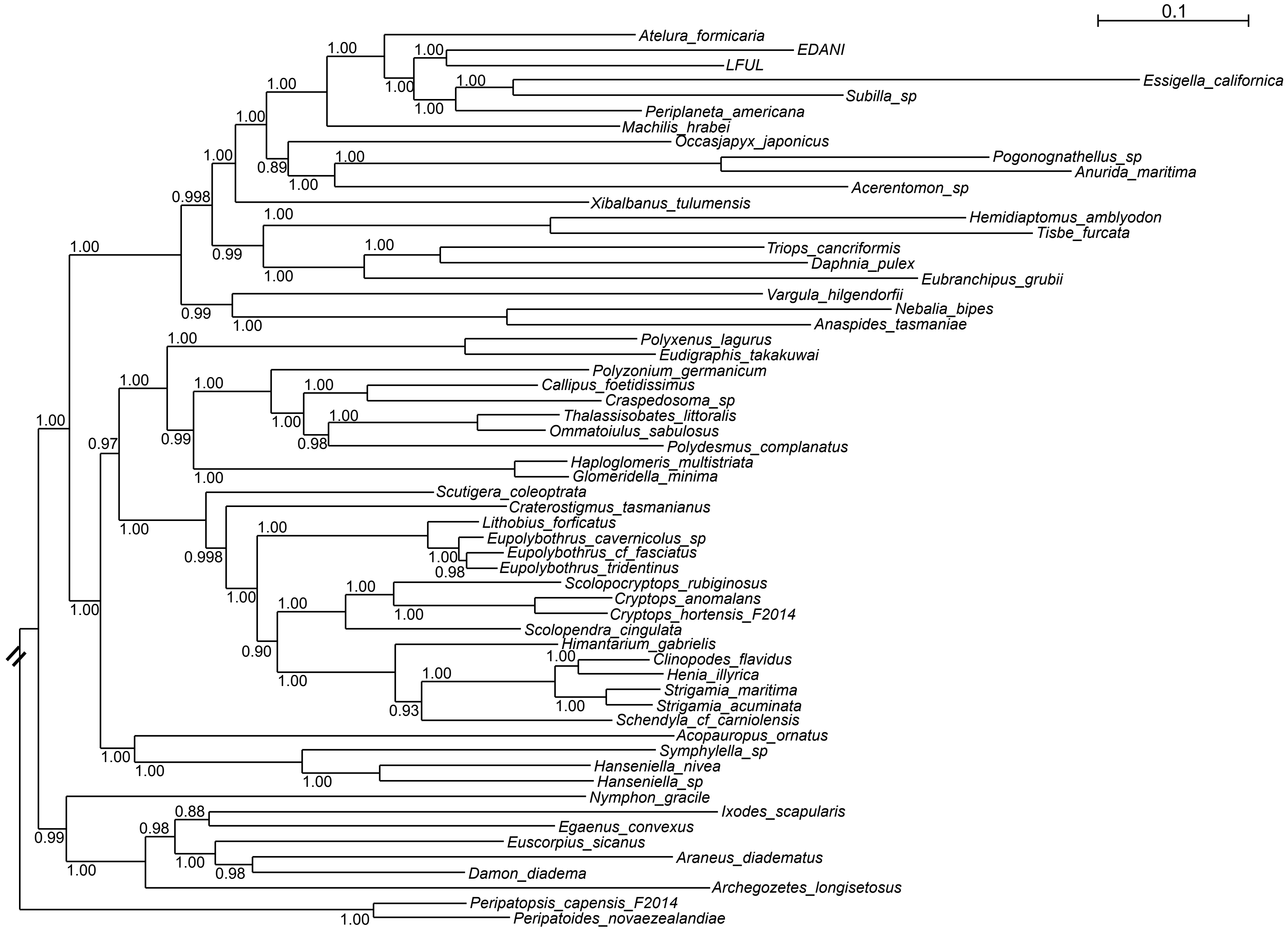

Fig. S7. Best ML tree inferred from the data set STRICTaa with transfer bootstrap support.
The ML tree is identical with the ML tree displayed in Fig. 2a with statistical transfer bootstrap support (TBE) inferred from all bootstrap trees with *Booster* v. 0.1.2. Values range from 0-1 (rounded to two decimal places). The tree was rooted with Onychophora.

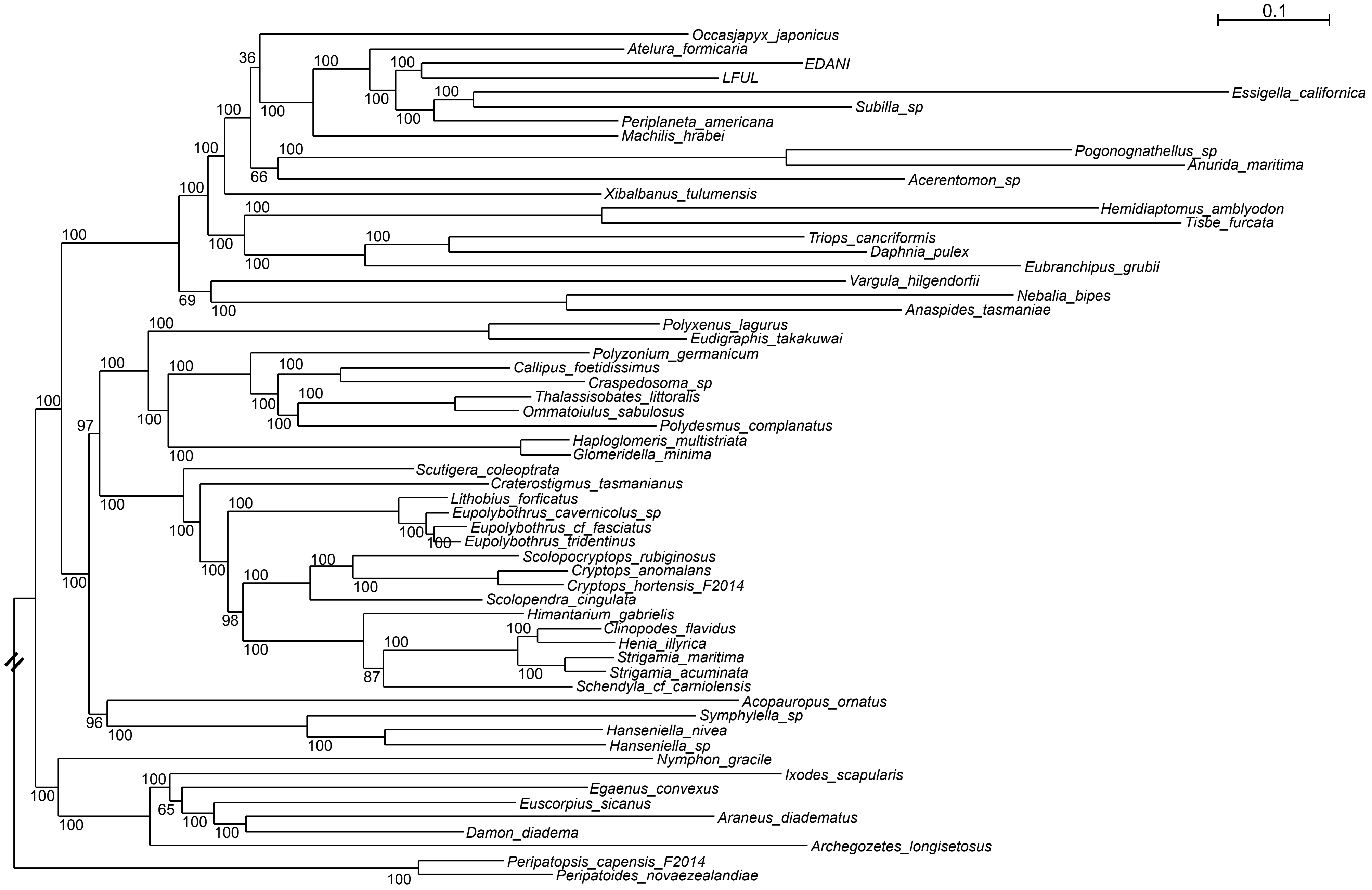

Fig. S8. Inferred ML tree from the data set STRICTaa with the CAT-like mixture model + PSMF.

Inferred ML tree from the data set STRICTaa using the unpartitioned approach applying the CAT-like mixture model + PSMF with statistical non-parametric bootstrap support inferred from 100 replicates. The tree was rooted with Onychophora.

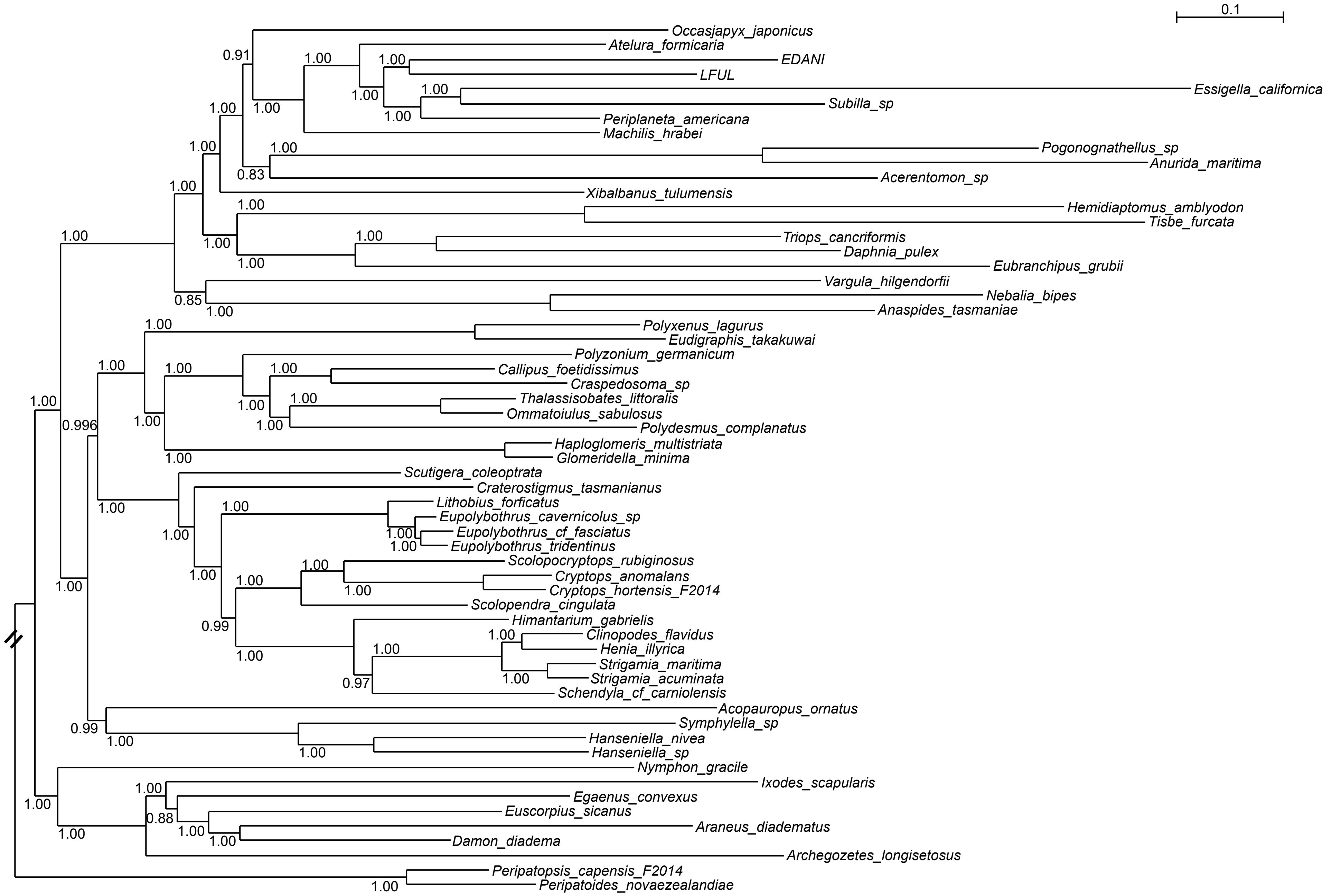

Fig. S9. Inferred ML tree from the data set STRICTaa with theCAT-like mixture model + PSMF with transfer bootstrap support. The ML tree is identical to the ML tree displayed in Fig. S8 with statistical transfer bootstrap support (TBE) inferred from all bootstrap trees with *Booster* v. 0.1.2. Values range from 0-1 (rounded to two decimal places).

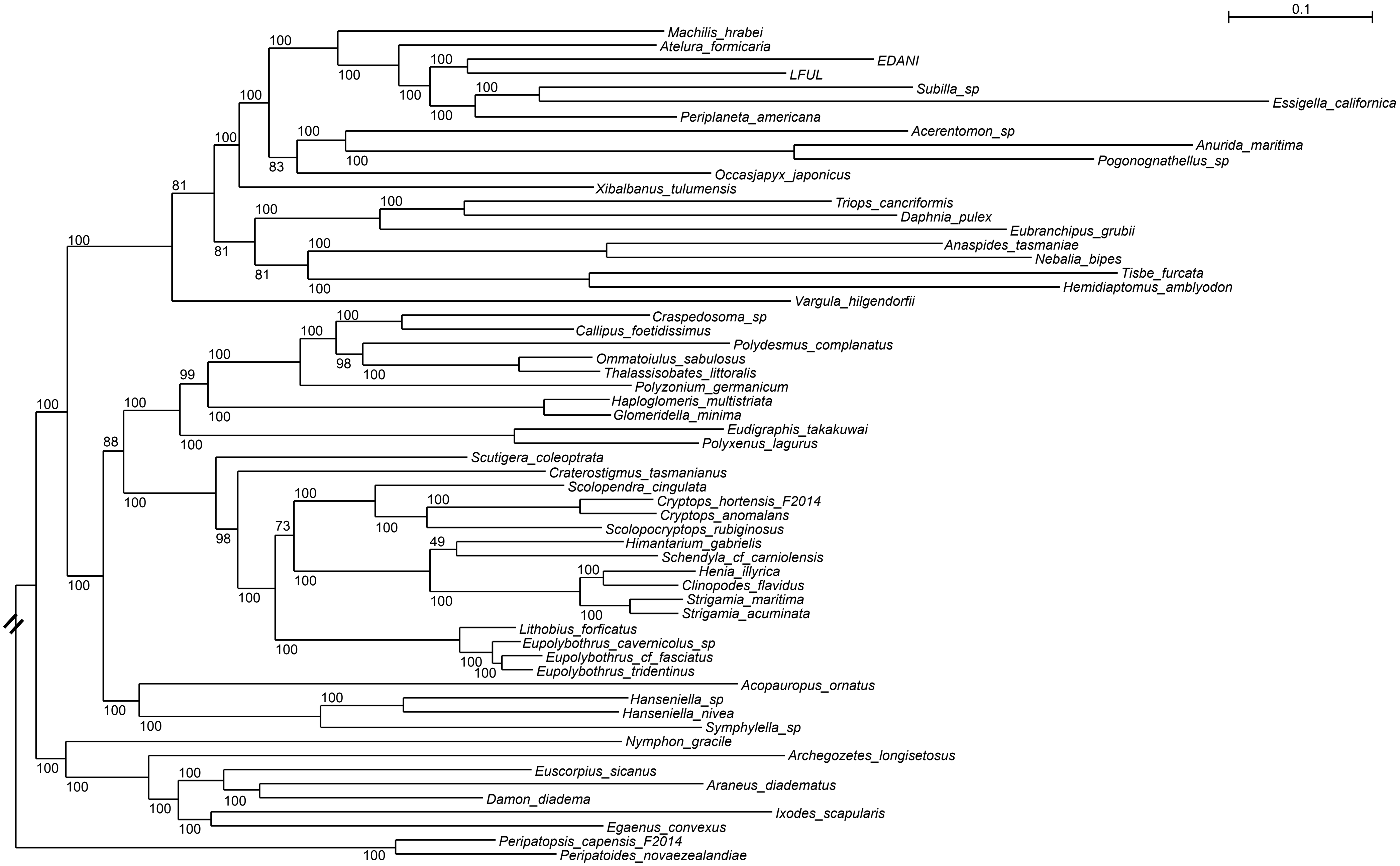

Fig. S10. Best ML tree inferred from the data set RELAXEDaa.
Statistical non-parametric bootstrap support was inferred from 100 replicates. The tree was rooted with Onychophora.

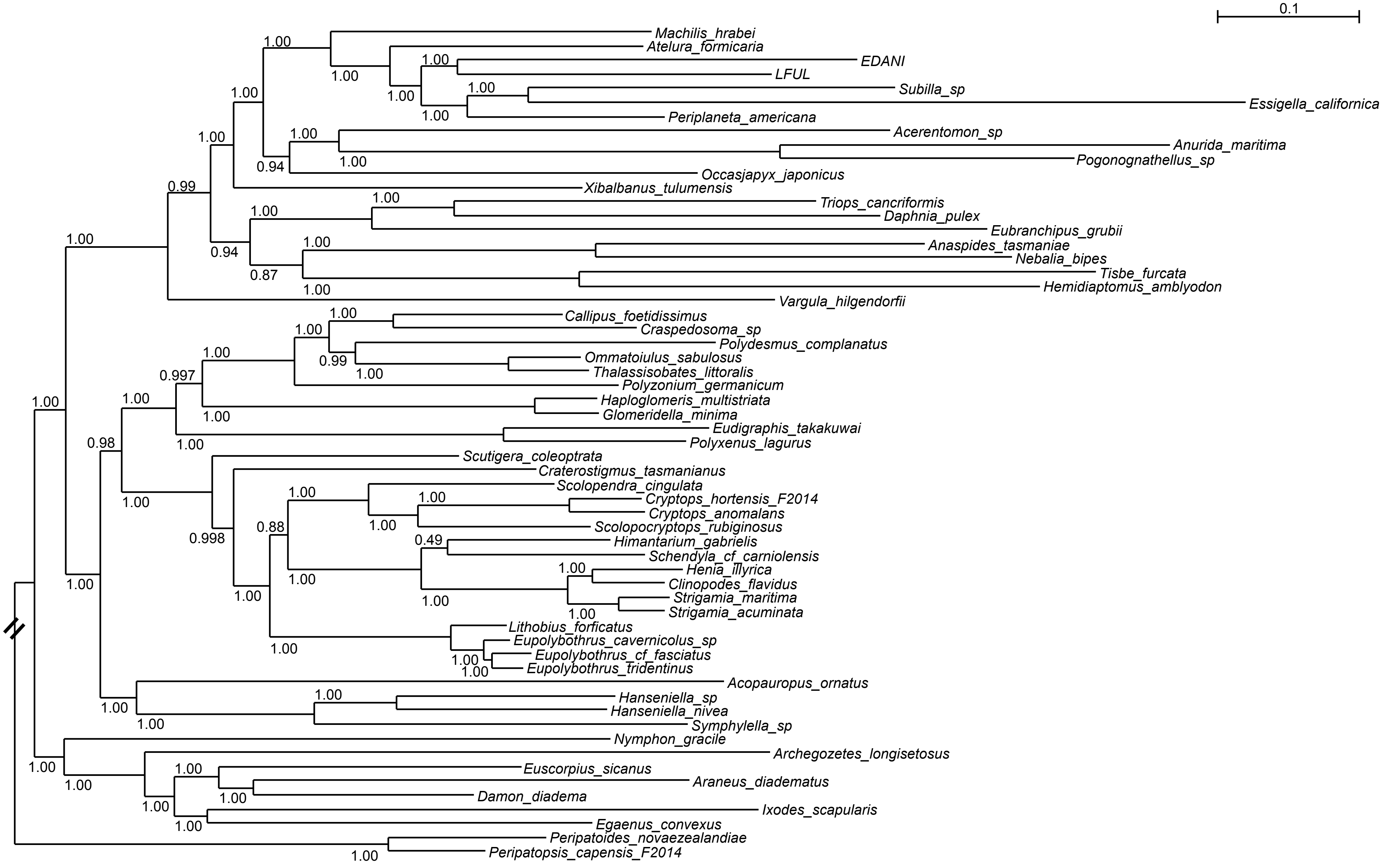

Fig. S11. Best ML tree inferred from the data set RELAXEDaa with transfer bootstrap support.
The ML tree is identical to the ML tree displayed in Fig. S10 with statistical transfer bootstrap support (TBE) inferred from all bootstrap trees with *Booster* v. 0.1.2. Values range from 0-1 (rounded to two decimal places). The tree was rooted with Onychophora.

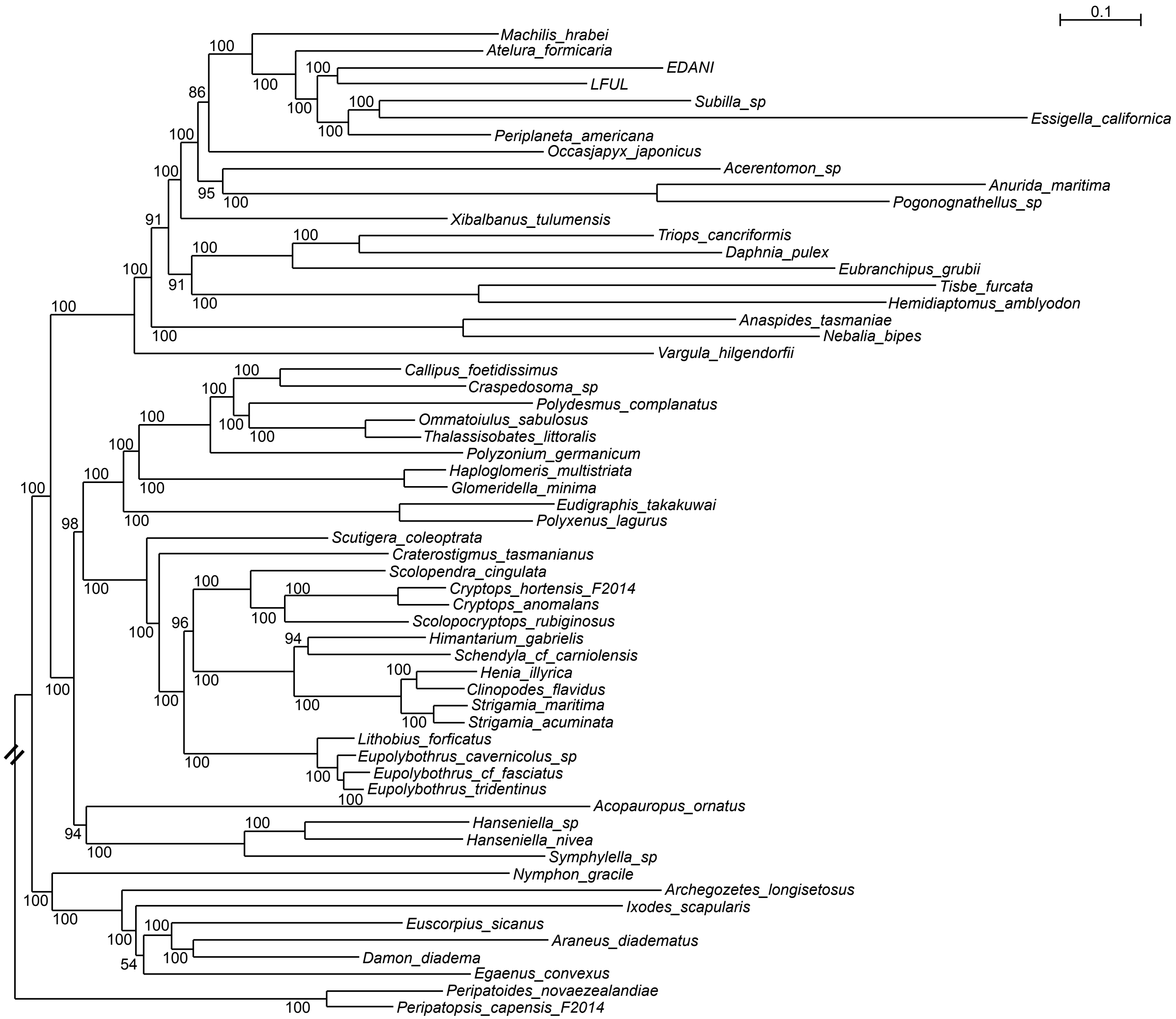

Fig. S12. Inferred ML tree from the data set RELAXEDaa with the CAT-like mixture model + PSMF.
Inferred ML tree from the data set RELAXEDaa using the unpartitioned approach applying the CAT-like mixture model + PSMF with statistical non-parametric bootstrap support inferred from 100 replicates. The tree was rooted with Onychophora.

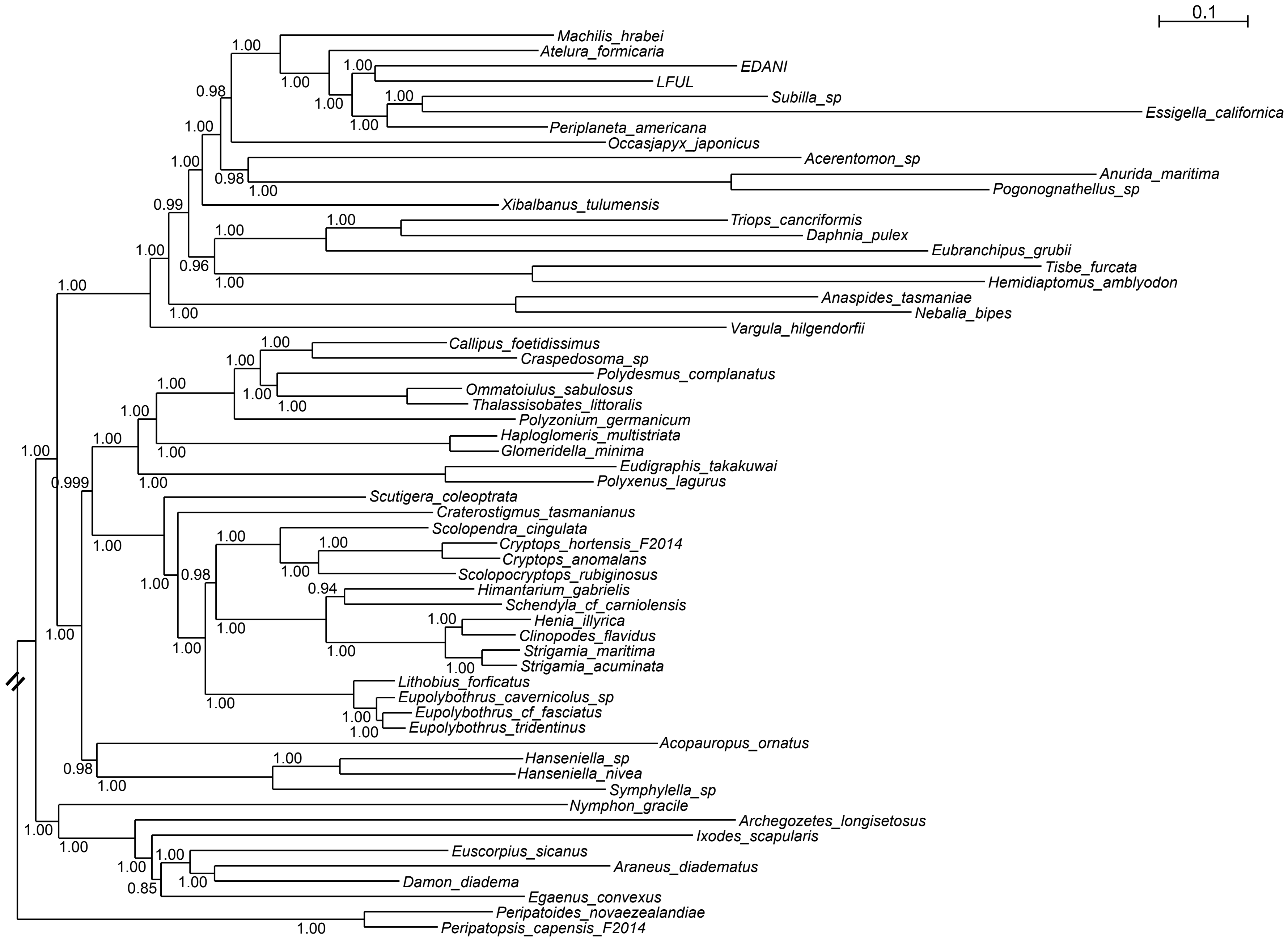

Fig. S13. Inferred ML tree from the data set RELAXEDaa with the CAT-like mixture model + PSMF with transfer bootstrap support. The ML tree is identical to the ML tree displayed in Fig. S12 with statistical transfer bootstrap support (TBE) inferred from all bootstrap trees with *Booster* v. 0.1.2. Values range from 0-1 (rounded to two decimal places). The tree was rooted with Onychophora.

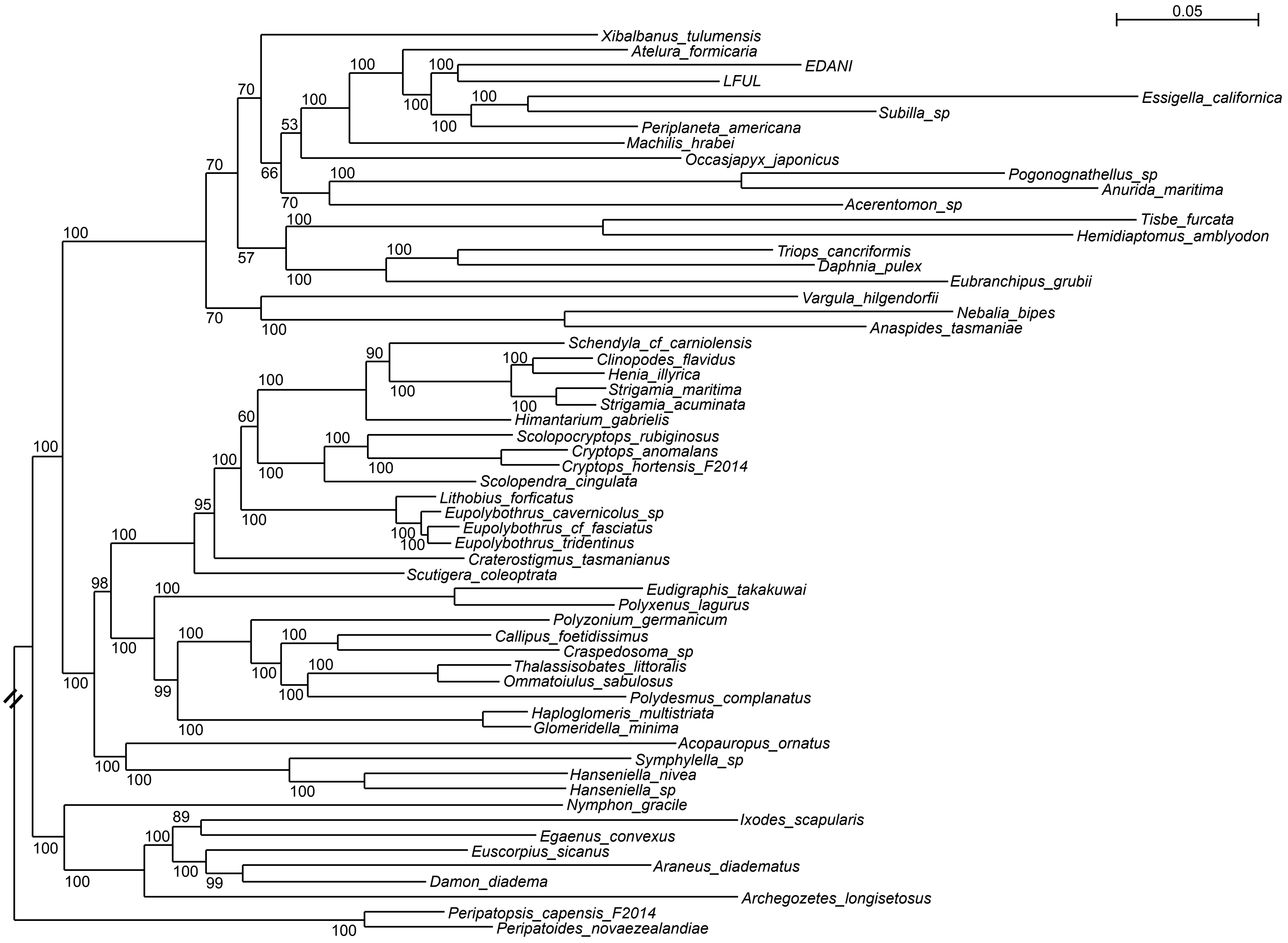

Fig. S14: Best ML tree inferred from the data set STRICTnt.
Data set STRICTnt only includes 2nd codon positions. Statistical non-parametric bootstrap support was inferred from 100 replicates. The tree was rooted with Onychophora.

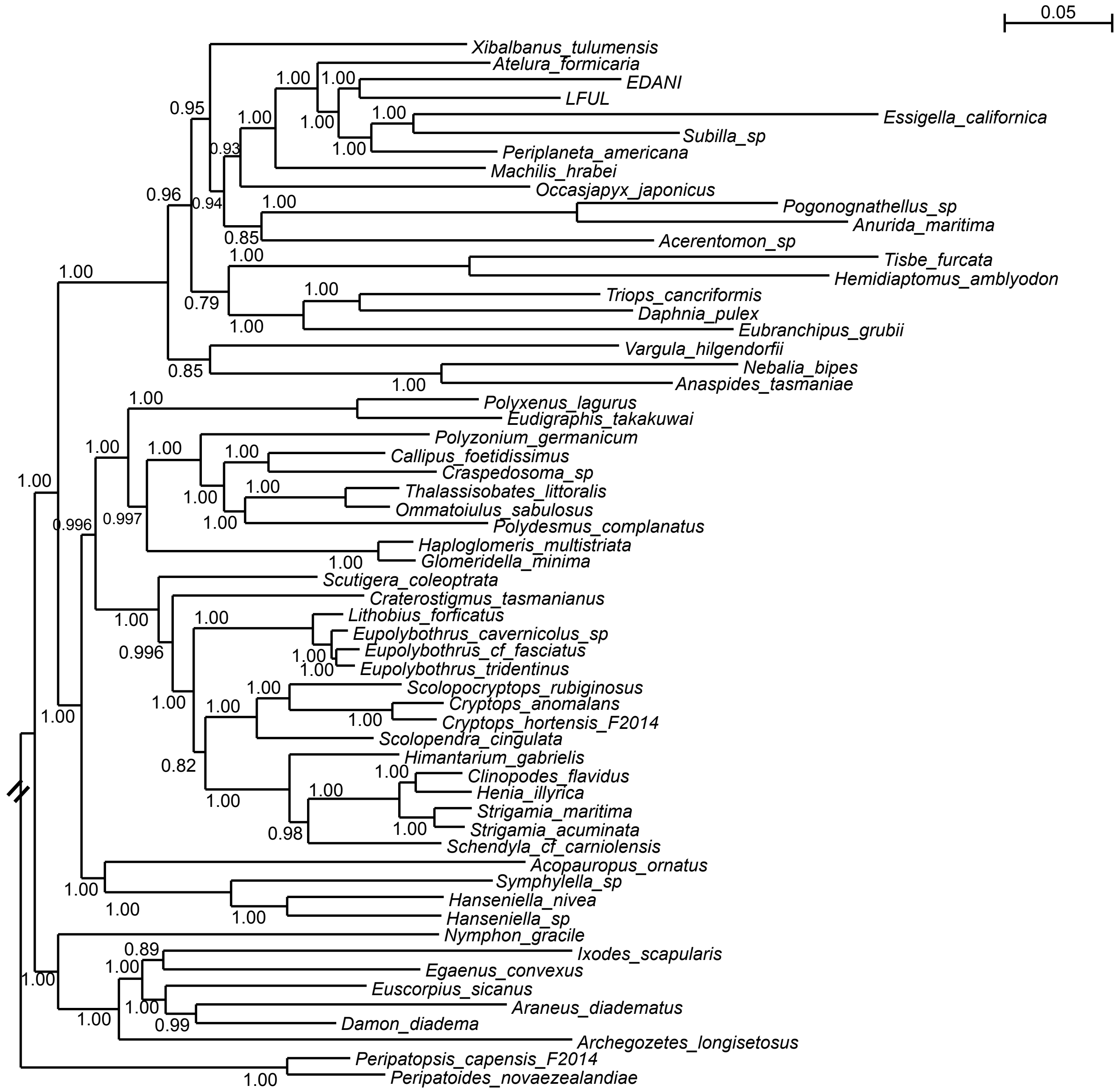

Fig. S15. Best ML tree inferred from the data set STRICTnt with transfer bootstrap support.
The ML tree is identical to the ML tree displayed in Fig. S14 with statistical transfer bootstrap support (TBE) inferred from all bootstrap trees with *Booster* v. 0.1.2. Values range from 0-1 (rounded to two decimal places). The tree was rooted with Onychophora.

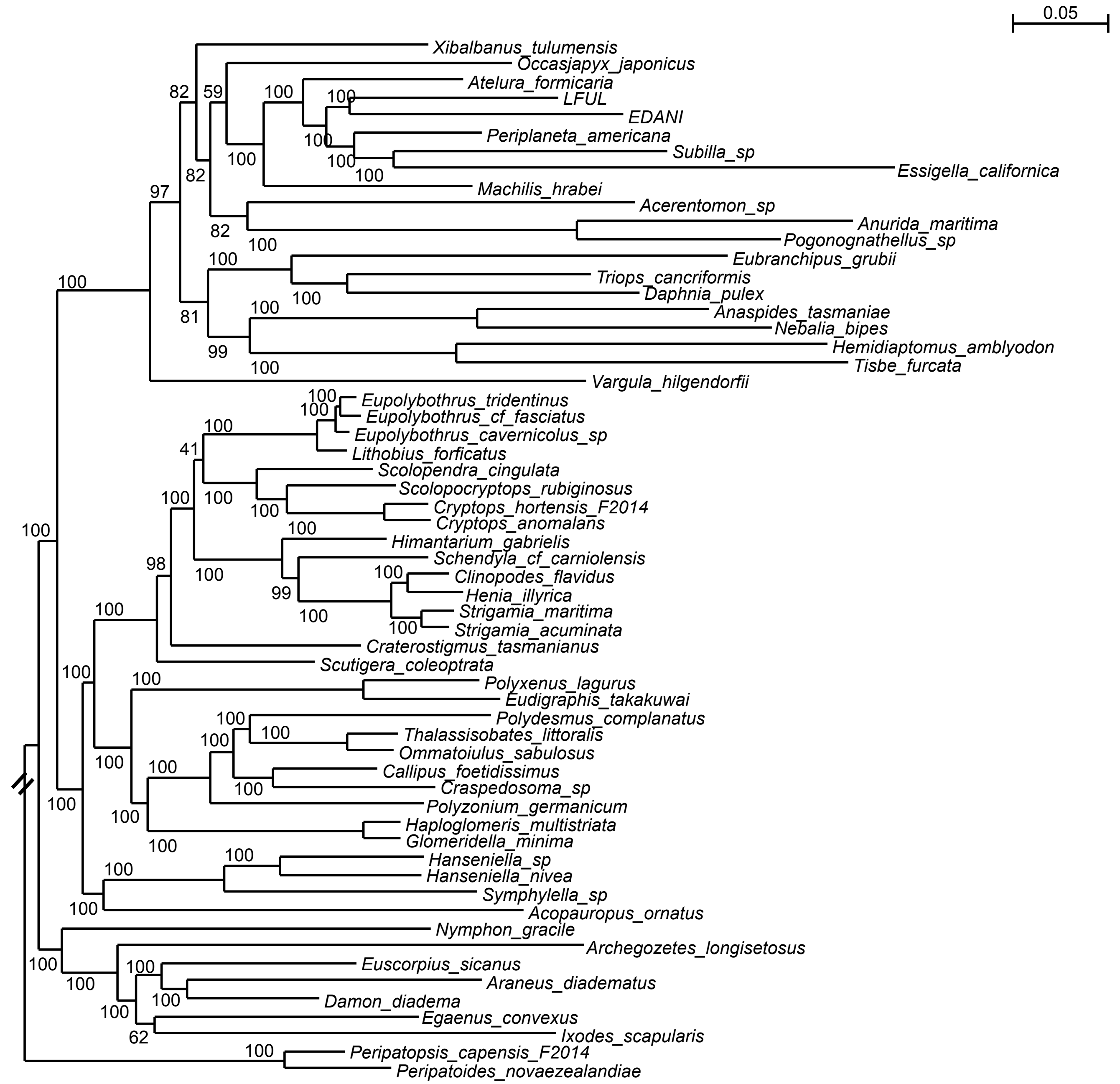

Fig. S16. Best ML tree inferred from the data set RELAXEDnt with non-parametric statistical bootstrap support. Data set RELAXEDnt only includes 2nd codon positions.Statistical non-parametric bootstrap support was inferred from 100 replicates. The tree was rooted with Onychophora.

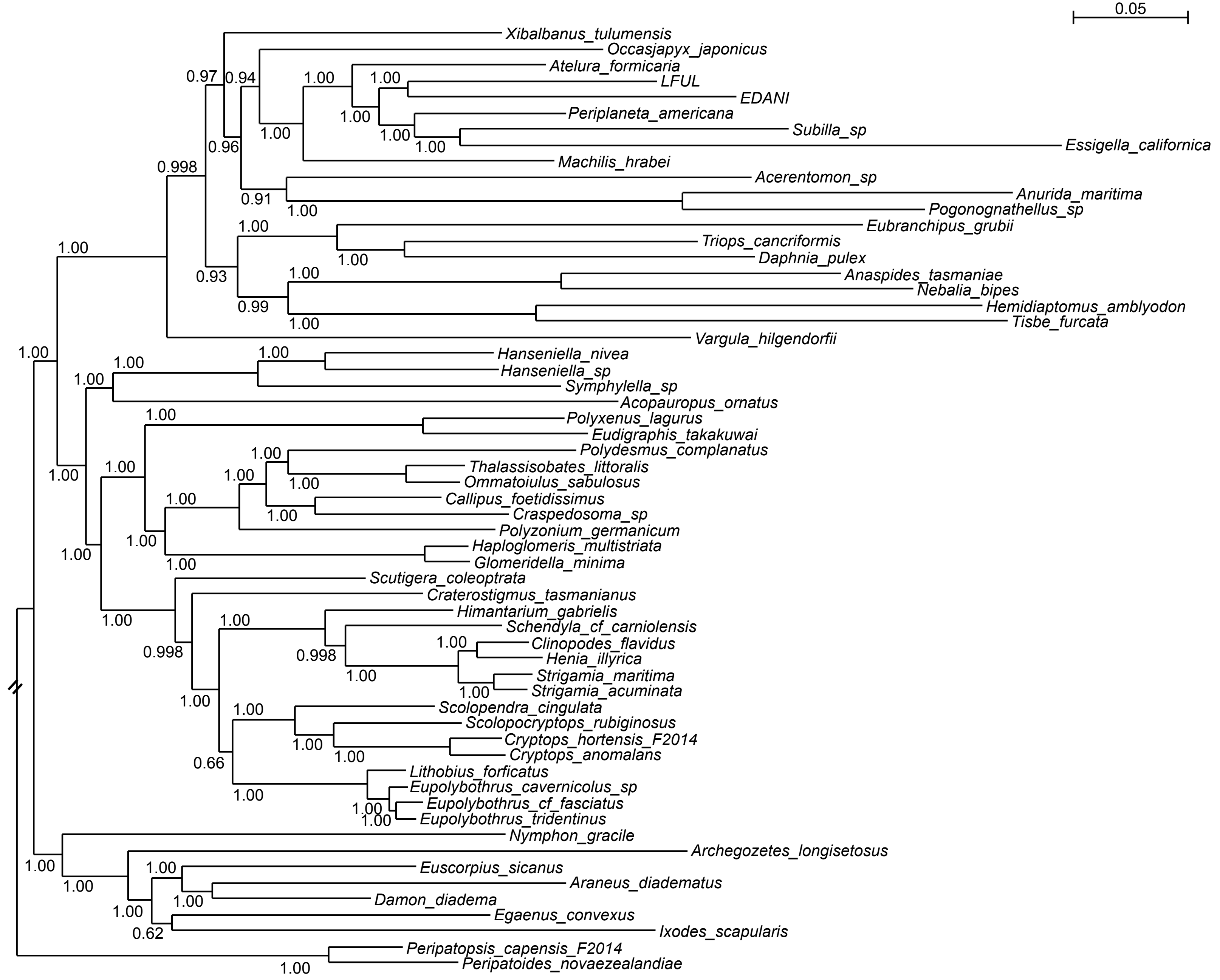

Fig. S17. Best ML tree inferred from the data set RELAXEDnt with transfer bootstrap support.
The ML tree is identical to the ML tree displayed in Fig. S16 with statistical transfer bootstrap support (TBE) inferred from all bootstrap trees with *Booster* v. 0.1.2. Values range from 0-1 (rounded to two decimal places). The tree was rooted with Onychophora.

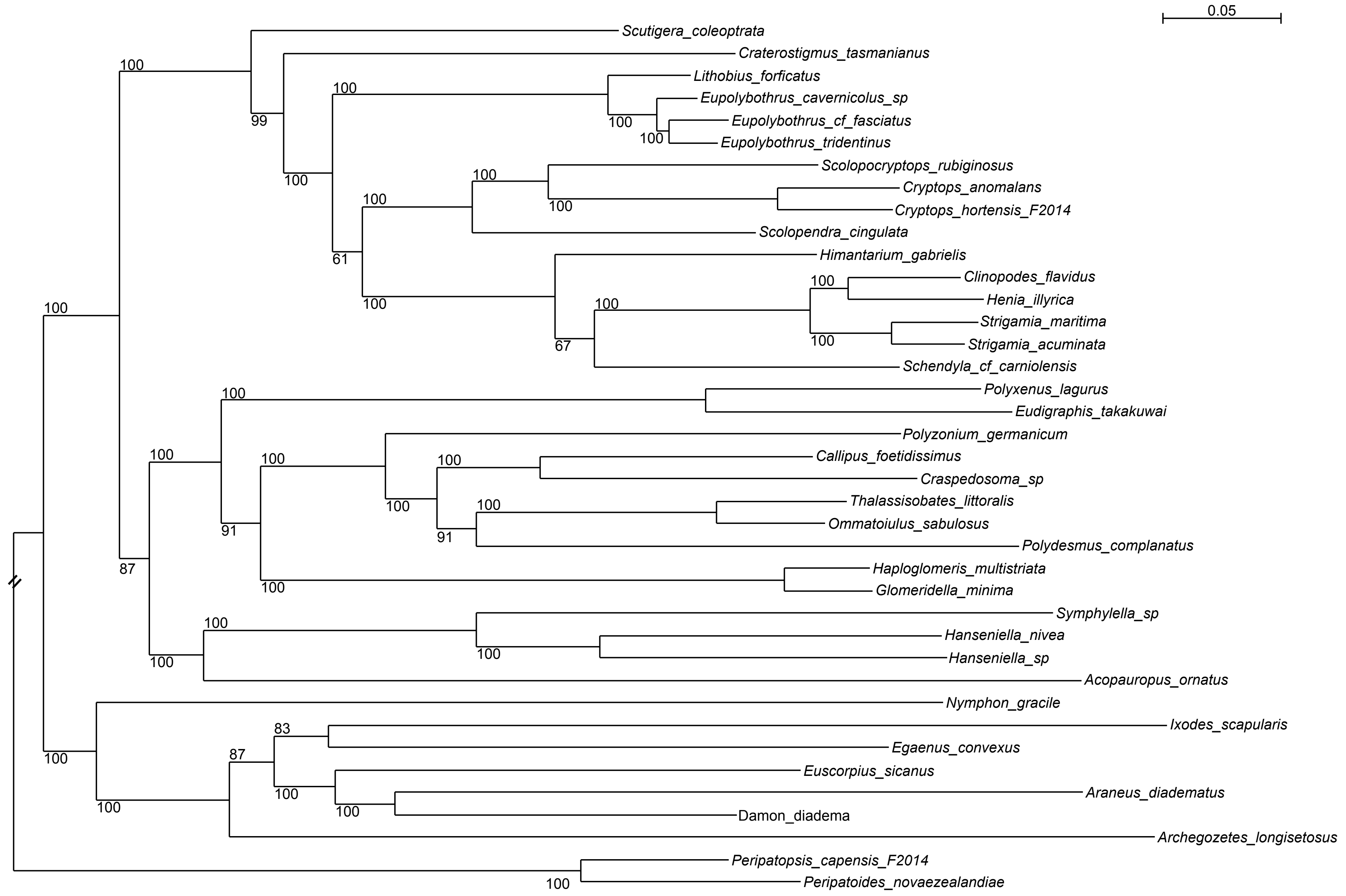

Fig. S18. Best ML tree inferred from the data set STRICTaa_ChO.
Data set STRICTaa_ChO includes only Chelicerata and Onychophora as outgroup (excluding Pancrustacea). Statistical non-parametric bootstrap support was inferred from 100 replicates. The tree was rooted with Onychophora.

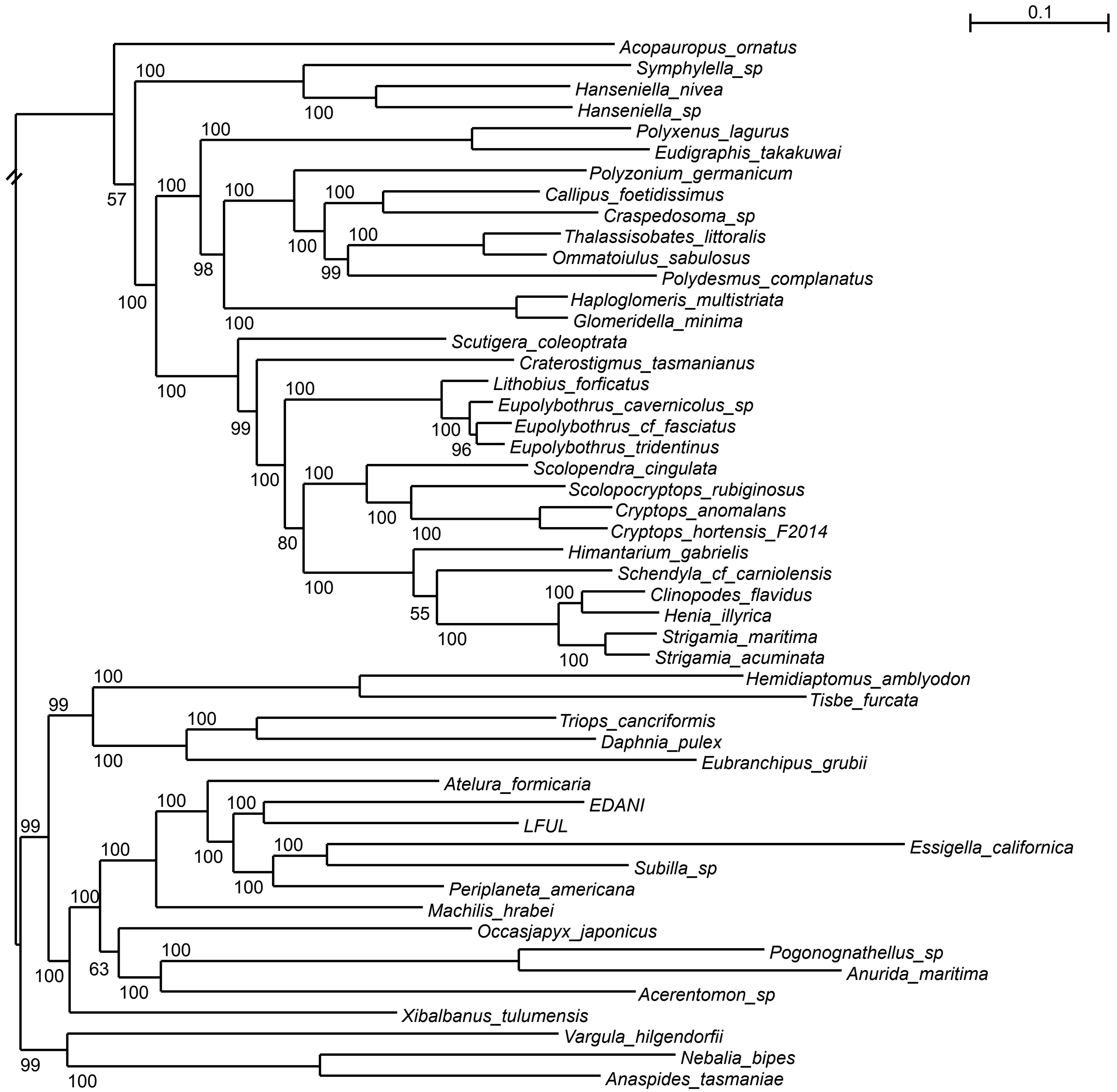

Fig. S19. Best ML tree inferred from the data set STRICTaa_Pan.
Data set STRICTaa_Pan includes only Pancrustacea as outgroup (excluding Chelicerata and Onychophora). Statistical non-parametric bootstrap support was inferred from 100 replicates. The tree was rooted with Pancrustacea.
